# Super-resolution imaging of platelet-activation process and its quantitative analysis

**DOI:** 10.1038/s41598-021-89799-9

**Published:** 2021-05-18

**Authors:** Jinkyoung Chung, Dokyung Jeong, Geun-ho Kim, Seokran Go, Jaewoo Song, Eunyoung Moon, Yang Hoon Huh, Doory Kim

**Affiliations:** 1 Department of Chemistry, Hanyang University, Seoul, 04763 Republic of Korea; 2 Department of Laboratory Medicine, Yonsei University College of Medicine, Seoul, 03722 Republic of Korea; 3 Electron Microscopy Research Center, Korea Basic Science Institute, Cheongju, 28119 Republic of Korea; 4 Research Institute for Convergence of Basic Sciences, Hanyang University, Seoul, 04763 Republic of Korea; 5 Institute of Nano Science and Technology, Hanyang University, Seoul, 04763 Republic of Korea; 6 Research Institute for Natural Sciences, Hanyang University, Seoul, 04763 Republic of Korea

**Keywords:** Biophysics, Cell biology

## Abstract

Understanding the platelet activation molecular pathways by characterizing specific protein clusters within platelets is essential to identify the platelet activation state and improve the existing therapies for hemostatic disorders. Here, we employed various state-of-the-art super-resolution imaging and quantification methods to characterize the platelet spatiotemporal ultrastructural change during the activation process due to phorbol 12-myristate 13-acetate (PMA) stimuli by observing the cytoskeletal elements and various organelles at nanoscale, which cannot be done using conventional microscopy. Platelets could be spread out with the guidance of actin and microtubules, and most organelles were centralized probably due to the limited space of the peripheral thin regions or the close association with the open canalicular system (OCS). Among the centralized organelles, we provided evidence that granules are fused with the OCS to release their cargo through enlarged OCS. These findings highlight the concerted ultrastructural reorganization and relative arrangements of various organelles upon activation and call for a reassessment of previously unresolved complex and multi-factorial activation processes.

## Introduction

Platelets are a blood component generated from megakaryocytes in the bone marrow. Their primary function is hemostasis, whereby platelets are activated at the damaged endothelium site, leading to the formation of fibrin-stabilized platelet aggregates, which are called a “platelet plug”. Abnormalities in this process are characterized by clinical bleeding, and many hemostatic disorders have been reported^1,2^. Therefore, investigating platelet activation pathways has been the focus of many studies, and a better understanding of the platelet activation mechanisms will undoubtedly be critical to improve various hemostatic disorder therapies^3^.

Platelet activation is a complex and multi-factorial process^4^. In response to injury, platelets rapidly adhere to the damaged blood vessel walls and interact with the exposed collagen and von Willebrand factor^5^. They can also be activated by the interaction of other agonists such as thrombin, arachidonic acid, and adenosine diphosphate (ADP) with the receptors expressed on the platelet surface or by activating the protein kinase C (PKC) family using phorbol 12-myristate 13-acetate (PMA)^6,7^. Additionally, inflammation also drives platelet activation, thus inducing the release of platelet-derived extracellular vesicles (PEVs) that is followed by the initiation of emergency hematopoiesis^8^. Endogenous proteins in activated platelets undergo a significant rearrangement with alterations in the number and size of their clusters, depending on their type and the induced activation type^9^. For example, the platelet factor 4 cluster shows a central distribution, whereas the fibrinogen clusters show a strong reallocation towards the periphery of the platelets upon ADP activation^9^. Thus, recent studies have demonstrated that the platelet activation process is far more complicated than previously thought due to an elaborate intracellular molecular machinery.

However, observation and quantification of the distribution of specific protein clusters in activated platelets still remains challenging because of their small dimensions (2–4 μm in diameter)^9^. The typical size of organelles in platelets is sub-diffraction-limited; thus, it is challenging to examine the distribution of proteins within platelets using conventional light microscopy^9,10^. Although electron microscopy (EM) can provide higher resolution, the embedded and sectioned samples are not thick enough to show the entire three-dimensional ultrastructure of organelles in platelets. It is therefore difficult to observe the complete protein content within platelets from TEM using thin sections. Recent advances in EM techniques, such as focused ion beam-scanning electron microscopy (FIB-SEM), serial block face-scanning electron microscopy (SBF‐SEM), and scanning transmission electron microscope (STEM) tomography, have allowed the 3D structures of organelles in platelets to be observed; however, imaging 3D full structures of large and complete volumes of activated and aggregated platelets remains challenging due to the sample thickness limit^10–15^. It is also difficult to identify organelles just from EM images because of the low protein specificity^16^. Thus, elucidating the specific molecular controls and pathways involved in platelet activation is an unfinished task, although the platelet activation basic mechanisms have been investigated.

These conventional microscopy limitations can be overcome by recent advances in super-resolution fluorescence microscopy^16–19^. Using stochastic optical reconstruction microscopy (STORM) imaging, we investigated the ultrastructural change of platelets at a nanoscale resolution during their activation process by observing the cytoskeletal elements and other organelles. To investigate the intracellular molecular mechanisms of organelles during the platelet activation process, the distribution and size of organelles at nanoscale were monitored and quantified. This advance overcomes the quantification limitations of conventional microscopy, and the resulting analysis of platelet ultrastructural changes can be key for understanding the molecular mechanisms that occurs during adhesion and activation. For comparison, we also performed live-cell three-dimensional optical diffraction tomography, live-cell fluorescence imaging, and various EM techniques, including scanning electron microscopy (SEM), transmission electron microscopy (TEM), correlative STORM and EM, and high-voltage transmission microscopy (HV-EM). Using these approaches, we investigated the ultrastructural change of platelets at the nanoscale during their activation process by observing the cytoskeletal elements and other organelles, including the internal membrane systems, such as the open canalicular system (OCS) and dense tubular system (DTS), which have not been resolved with conventional microscopy. We present the detailed roles and interactions between organelles during the platelet activation process. We reveal the ultrastructural arrangements, along with their quantitative timeline, that were not resolved in previous experiments.

## Results

### Live-cell imaging of activated platelets

**Figure 1:**
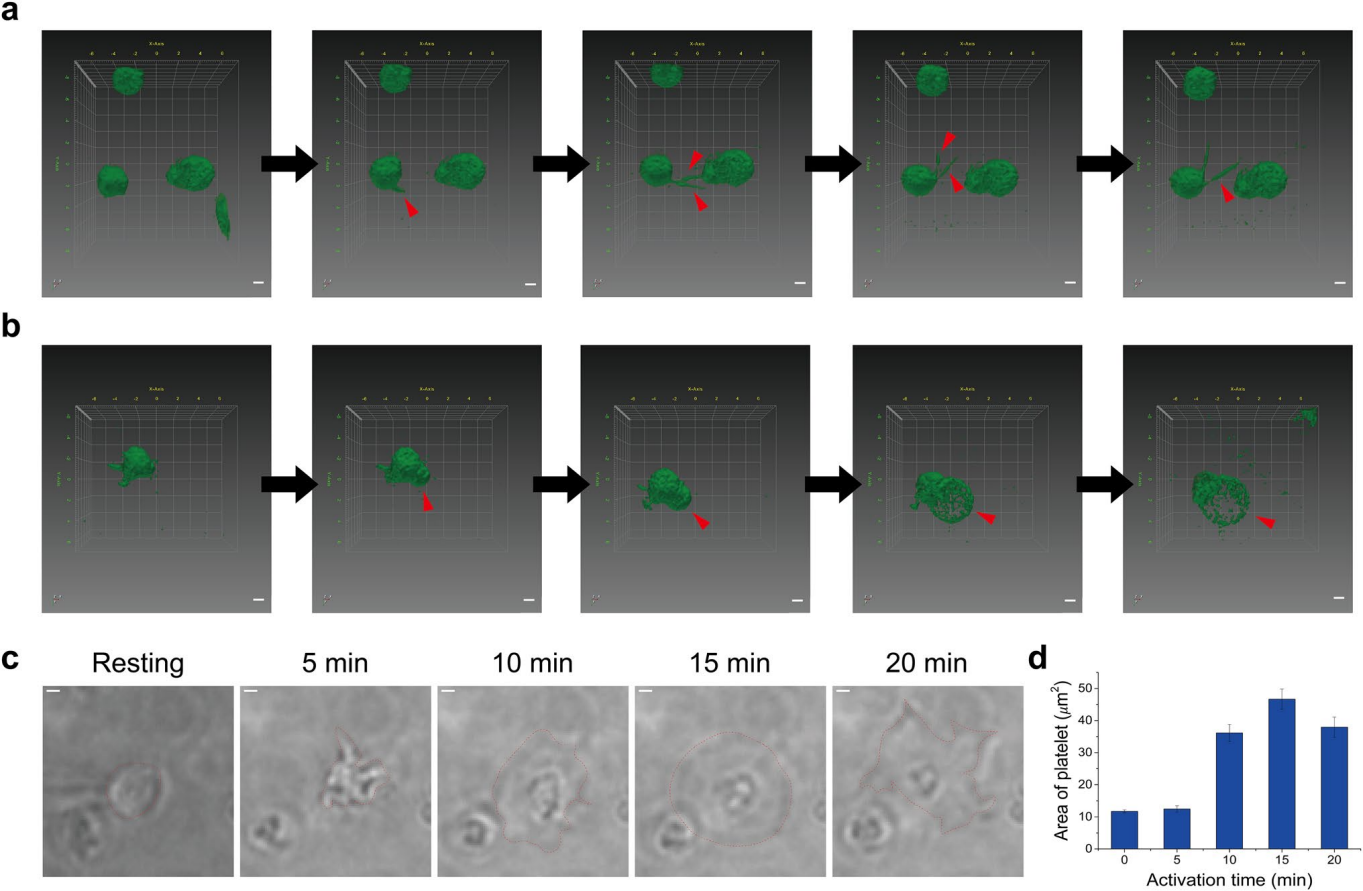
Spatiotemporal dynamics of live platelets on a glass coverslip. (**a**) 3D optical diffraction tomography time-lapse sequence showing a platelet with dynamic protrusions (red arrow) for sensing the environment as the first step of platelet activation (5 min per snapshot). (**b**) 3D optical diffraction tomography time-lapse sequence showing a platelet undergoing ballooning (red arrow) on adhesion to a glass surface as the first step of platelet activation (5 min per snapshot). (**c**) Examples of activated platelets showing five consecutive differential-interference-contrast snapshots (5 min per snapshot). The red dashed line represents the boundary of the platelet identified from the DIC images. (**d**) The bar graph indicating the area change of a platelet upon activation induced by PMA (mean ± SD; n = 34–60). Scale bars 1 μm in (**a**–**c**).

We started with live-cell imaging using three-dimensional optical diffraction tomography to observe the initial state of platelets during the early adhesion process on the coverglass. The 3D holographic videos of living platelets reveal that a few platelets are highly dynamic upon adhesion onto the coverglass, exhibiting protrusions from platelets, as shown in Fig. 1a and Supplementary Video 1. These highly dynamic protrusions are likely to probe the environment as sensors to find anchors on the glass coverslip for spreading. We also often observed ballooning phenomena of platelets, as shown in Fig. 1b and Supplementary Video 2. Platelet balloon formation and extending pseudopodia are known to be part of the normal hemostatic response, and they were observed in the early phases of contact with the glass coverslip under our experimental conditions^20,21^. Therefore, it is likely that platelets can initiate their activation processes upon contact with the coverglass, even without any agonist. However, this activation process was too slow to be observed without the use of an agonist. (Supplementary Fig. S1).

The activation process has been previously investigated via treatments using various agonists (e.g., ADP or thrombin) or PMA to expedite this process. Among these, PMA is a diacylglycerol analog that is known to induce platelet aggregation through the activation of protein kinase C in platelets, and this is associated with the release of serotonin from platelets^22–24^. Although it is known that structural changes in platelets can vary depending on the type of activation induced, little is known about the ultrastructural changes of endogenous proteins in activated platelets induced by PMA^9^. Therefore, we decided to investigate this effect by inducing the activation of platelets using PMA. To analyze the changes in the overall platelet shape induced by PMA, we performed differential-interference-contrast (DIC) imaging of the platelets treated with PMA. We found that the resting platelets shifted from a discoid-shape to exhibiting long pseudopodia after a few minutes of PMA treatment, and then spread on the glass coverslip, which is consistent with previous studies (Fig. 1c)^25^. The area of platelets increased ~ 10 to 50 μm^2^ during activation due to the spreading effect (Fig. 1d).

### Super-resolution imaging of main cytoskeleton elements in activated platelets

**Figure 2:**
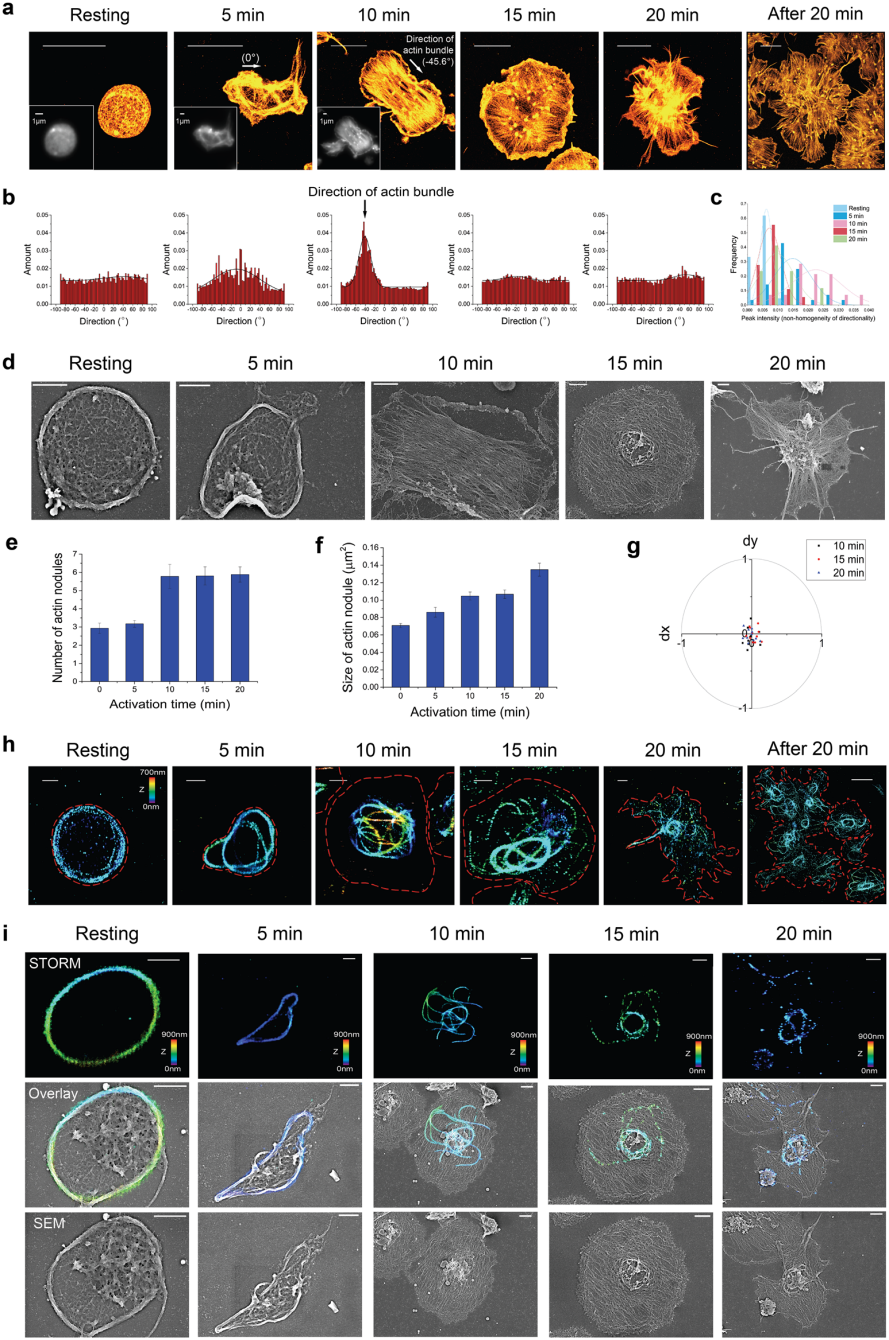
Super-resolution images of main cytoskeleton elements in activated platelets. (**a**) 2D STORM images of actin filaments in activated platelets. Actin filaments were observed from the platelets that were fixed at different activation time points (0, 5, 10, 15, and 20 min). After 20 min, the activated platelets were aggregated. (**b**) The directionality histogram indicating the amount of structures in a given direction to infer the preferred orientation of structures from the STORM image shown in (**a**). (**c**) The distribution of directional non-homogeneity at different activation time points (n = 14–28). (**d**) SEM images of activated platelets. (**e**,**f**) The bar graph indicating the number (**e**) and size (**f**) of actin node in a platelet during the activation process observed from STORM images (mean ± SD; n = 23–61). (**g**) The relative position difference (dx, dy) between the center of mass of nodules and the centroid of the spread platelet for activated platelets (10, 15, 20 min), showing their similar values (n = 10–12). (**h**) 3D STORM images of microtubules in the activated platelet. Microtubules were observed from the platelets that were fixed at different activation time points (0, 5, 10, 15, and 20 min). The red dashed line represents the boundary of the platelet identified from the corresponding DIC images (white arrows: formation of the small microtubule ring). (**i**) 2D and 3D STORM images of the resting (0 min) (left) and activated (15 min) platelets (right) stained for acetylated microtubules. The red dashed line represents the boundary of the platelet identified from the corresponding DIC images. (**j**) Correlative STORM and SEM images of microtubules in the activated platelets fixed at different activation time points (0, 5, 10, 15, and 20 min). Top: STORM; middle: overlay; bottom: SEM (white arrows: formation of the small microtubule ring; yellow arrow: pseudopodia, probably consisting of actin filaments). Scale bar 5 μm in (**a**) and 1 μm in (**d**,**h**,**i**,**j**).

To investigate the cytoskeletal changes in platelets at the ultrastructural level during the activation process, we started with STORM imaging of actin filaments. Individual actin filaments were resolved particularly within the meshworks near the platelet boundary in the STORM images of fixed platelets, and they were distributed on the overall shape of platelets during the activation process (Fig. 2a, Supplementary Fig. S2a). In a resting platelet, homogeneously distributed mesh networks of actin were observed without actin bundles. After 5 min of PMA treatment, actin filaments started to exhibit protrusions, which seemed to probe the environment for spreading, as sensors. Protrusion formation is the initial morphological change associated with adhesion and aggregation^26^. Then, they started to spread out in one direction with the formation of actin bundles after 10 min of PMA treatment. This linear spreading direction was changed to a spreading in a radial fashion in 15 min, and then, the fully spread round shape of platelets exhibited newly formed protrusions in 20 min, which seemed to aid adherence to other platelets for aggregation (Supplementary Fig. S3). To demonstrate the directionality of spreading, we analyzed the amount of structures in a given direction from STORM images using the ‘directionality’ plugin of ImageJ and plot the histograms. As shown in Fig. 2b,c, the STORM images for 0 and 15 min showed flat histograms, which is interpreted as a complete isotropic spreading direction, whereas the STORM images for 5 and 10 min showed a histogram with a peak at the orientations of 0° and − 45.6°, respectively, which implies that there is a linear orientation.

We also performed SEM imaging to observe the exposed cytoskeletal elements, for comparison (Fig. 2d). From the SEM images, we could observe similar features of actin filaments as those observed in the STORM images during the activation process. Thus, actin is likely to be essential for platelet morphology maintenance. The thick peripheral ring structure in resting platelets was observed using SEM, likely representing well-known microtubule bundles. From both STORM and SEM images, we also observed that actin nodules were formed upon platelet activation. The number and size of actin nodules in a platelet increases during the activation process, probably due to an increased actin polymerization, which is in agreement with previous observations (Fig. 2e, f)^3^. The average number of actin nodules per activated platelet was six, with an average diameter of 410 nm. When we compared the center of mass of nodules with the centroid of the spread platelet, they exhibited similar values, implying that activated platelets can be spread out from the nodules as adhesion points (Fig. 2g). They share some similarities with podosomes, which are short-lived and F-actin structures enriched in integrins, Src family kinases, and the ARP2/3 complex in megakaryocytes^3^. The observed actin nodules exhibited a strong resemblance to the podosomes in dendritic cells that are interconnected by actin bundles radiating from the dense actin core^27^. Actin nodules are known to result from increased actin polymerization and play roles in mechano-coupling between the substrate and platelets and in platelet–platelet interaction^3^. Also, it is known that their absence could contribute to the pathological bleeding of Wiskott–Aldrich syndrome, which implies the role of actin nodules in platelet activation^3^. Thus, these results support the essential role of actin nodules and bundles in the activation process of platelets.

Next, we examined the ultrastructural organization changes of the microtubules during the activation process using 3D STORM (Fig. 2h, Supplementary Fig. S2b). In a resting platelet, the discoid shape of a platelet is maintained due to the organization of the microtubules in a ring structure in the periphery of platelets, which is called the marginal band (MB). These microtubule bundles started to unwind to individual microtubule filaments within 5 min of the PMA treatment. Since previous studies suggested that dynein is primarily responsible for sliding microtubules relative to one another in proplatelets, the observed unwinding MB could also be caused by the dynein-mediated microtubule-sliding process^28^. Fully unwinding microtubule filaments were observed within 10 min, and they started to form a smaller microtubular ring after 15 min, followed by the acquirement of the so-called ‘fried-egg’ morphology of spread platelets. At 20 min, the microtubules outside the small microtubular ring seemed to be depolymerized. We also performed STORM imaging of acetylated microtubules, and we found that these were mainly present in the small microtubular ring and not in the depolymerized microtubules outside of the small ring (Fig. 2i, Supplementary Fig. S2c).

To observe the relative localization of microtubules in detail inside the activated platelets, we performed the recently developed correlative STORM and SEM imaging (Fig. 2j). Since the unwinding of MB started with the spreading events upon activation, the larger volume outside the MB is likely to induce an imbalance between the microtubule elastic bending force and the bundling force, thus resulting in an unwinding process. From the correlative images, we found that the peripheral microtubules moved to the central region of activated platelets. We also found that microtubule filaments do not form pseudopodia, which probably mainly consist of actin filaments. Overall, we observed the reorganization of microtubules with their continued dynamic assembly and disassembly during the activation process.

**Figure 3:**
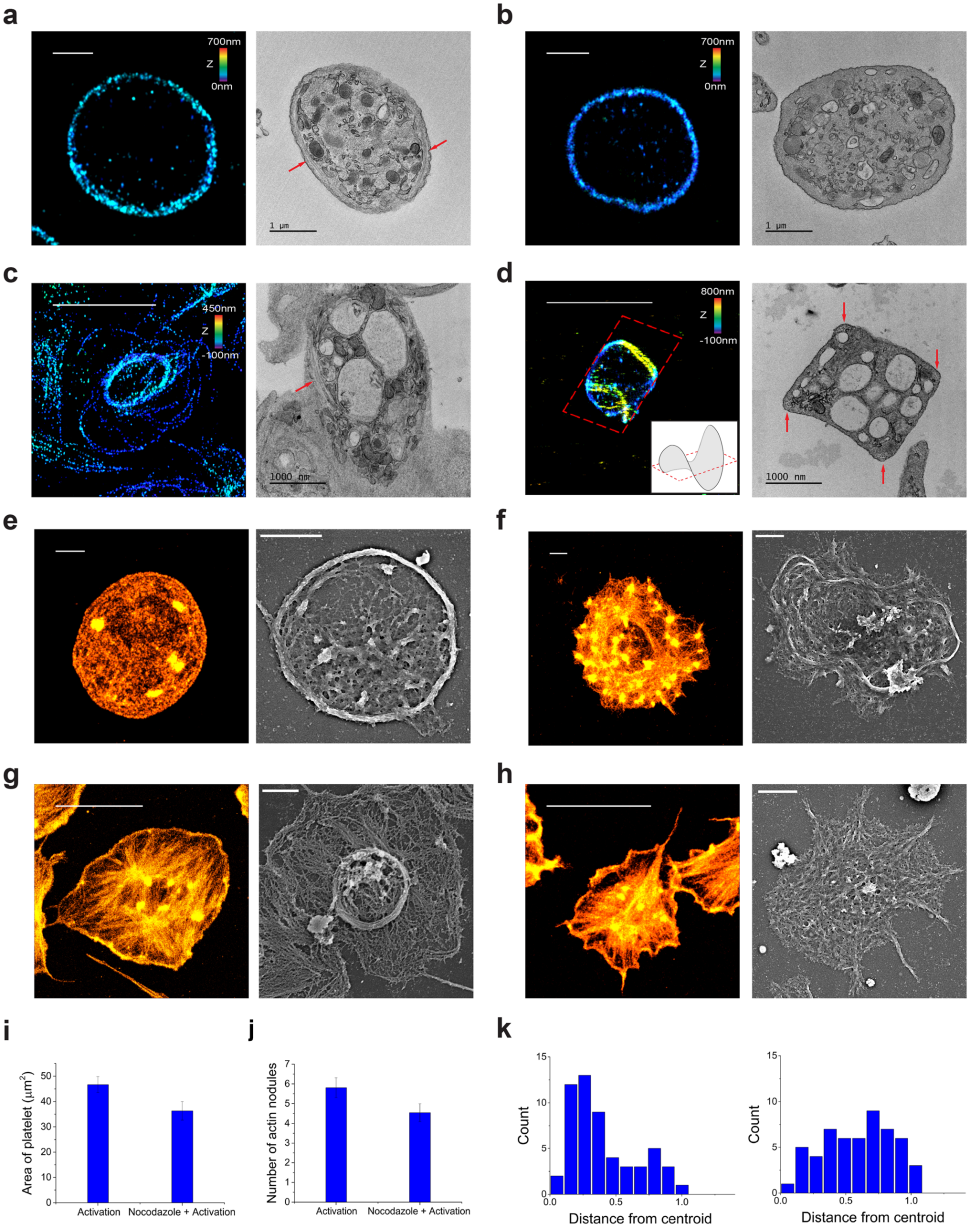
Effect of cytochalasin D and nocodazole pretreatment on ultrastructure of activated platelets. (**a**) Representative 3D STORM (left) and TEM (right) images of microtubules showing the MB in the resting platelet (red arrow: microtubules). (**b**) Representative 3D STORM (left) and TEM (right) images of microtubules in cytochalasin-D-pretreated resting platelets showing similar structure of microtubules to that in the control sample. (**c**) Representative 3D STORM (left) and TEM (right) images of microtubules showing the newly formed small ring of microtubules in the activated platelet (red arrow: microtubules). (**d**) Representative 3D STORM (left) and TEM (right) images of microtubules showing the ‘potato-chip’-like structure of microtubules in cytochalasin-D-pretreated activated platelets. Inset in 3D STORM image: schematic representation showing ‘potato-chip-like platelet’. TEM images of the cytochalasin D-pretreated activated platelet showing the microtubule bundles at four corners of the ‘potato chip’-like structure of microtubules (red arrow). (**e**) Representative 2D STORM (left) and SEM (right) images of actin filaments in a resting platelet. (**f**) Representative 2D STORM (left) and SEM (right) images of actin filaments in nocodazole-pretreated a resting platelet. (**g**) Representative 3D STORM (left) and SEM (right) images of actin filaments showing the radially spreading actin bundles with centralized actin nodules and a small ring of microtubules in activated platelets. (**h**) Representative 3D STORM (left) and SEM (right) images of actin filaments showing the radially spreading actin bundles with relatively dispersed actin nodules in the absence of a small ring of microtubules in nocodazole-pretreated activated platelets. (**i**) The bar graph indicates the area of non-treated and nocodazole-pretreated activated platelets (mean ± SD; n = 32, 40). (**j**) The bar graph indicates the number of actin nodules in non-treated and nocodazole-pretreated activated platelets (mean ± SD; n = 33, 40). (**k**) The radial distribution graph of actin nodules in non-treated (left) and nocodazole-pretreated (right) activated platelets from the STORM images shown in (**g**,**h**), showing moved nodules slightly towards the edge of the spread platelets, compared to non-treated samples. Scale bar 5 μm in (**c**,**d**,**g**,**h**) and 1 μm in (**a**,**b**,**e**,**f**).

Our cytoskeletal elements findings raise important questions regarding the role of actin filaments and microtubules in platelet activation. To further examine these roles, we investigated the effect of cytochalasin D and nocodazole pretreatments on the overall shapes of activated platelets, which disrupt actin and microtubules, respectively (Fig. 3). The complete depolymerization for actin and microtubules by cytochalasin D and nocodazole, respectively, was noted within 15 min (Supplementary Fig. S4). To observe their ultrastructural changes, we performed STORM and TEM imaging. First, when a resting platelet is treated with cytochalasin D, the platelets still show the discoid shape of MB without significant changes in their morphology. To investigate the role of actin in the activation process, the cytochalasin D-pretreated platelets were activated using 0.1 µg/mL PMA. Interestingly, we observed the square shape of relatively small platelets from TEM images, which has not been observed either from untreated resting or activated platelets (Fig. 3a, b, Supplementary Fig. S5). Interestingly, microtubule bundles were observed at the four corners of the square-shaped activated platelets. Since it was difficult to imagine the full three-dimensional structure of cytochalasin-D-pretreated platelets just from TEM section images, we performed 3D STORM imaging of the cytochalasin-D-pretreated activated platelets to investigate their entire structure (Fig. 3c,d). We found that this square shape of the sectioned platelet came from the ‘potato-chip-like structure’ of the cytochalasin-D-pretreated activated platelets. Actin depolymerized platelets could not be spread out on the coverglass upon activation; hence, MB could not unwind owing to the limited space, but it bent with flexibility inside the small volume of the activated platelet.

Next, we investigated the effect of nocodazole on resting platelets using STORM imaging. With the disruption of microtubules, we observed the formation of actin nodules with an increased platelet area due to a slight spreading effect (Fig. 3e,f). This result suggests that MB may play a role in inhibiting platelet spreading and maintaining the discoid shape of resting platelets by generating contractile forces. Meanwhile, the activation of the nocodazole-pretreated platelets, did not show significant differences in the overall morphology compared to normal activated platelets, which is in agreement with previous observations (Fig. 3g,h). However, from the quantification of the area of nocodazole-pretreated activated platelets, we found that they were less spread out under the nocodazole treatment (~ 78%) with the smaller number of actin nodules compared to non-treated activated platelets (Fig. 3i,j). Moreover, from the actin nodule distribution analysis, we observed that the nodules moved slightly towards the edge of the spread platelets, rather than being centralized with their reduced number, compared to non-treated samples (Fig. 3k). These results imply that microtubules may not play a significant role in controlling the overall structural shape changes of platelets but may inhibit or help the spreading process by arranging the actin nodes during the activation process. Overall, it is likely that actin is responsible for providing the spreading forces of activated platelets with the help of microtubules, and microtubules can control the extent of the spreading process with increased flexibility during the activation process.

We also performed 3D STORM imaging to elucidate the ultrastructural changes of the other cytoskeleton components during the activation process, including spectrin and vimentin (Supplementary Fig. S6). Unlike actin and microtubules, these components exhibited punctate and speckled patterns with uniform distribution on the surface of resting platelets. Upon activation, they were concentrated in the center of the activated platelets at a relatively high position and likely probably localized in the 'yolk’ spot of the ‘fried egg’ morphology of activated platelets.

### Super-resolution imaging of double membrane-bound organelles in activated platelets

**Figure 4:**
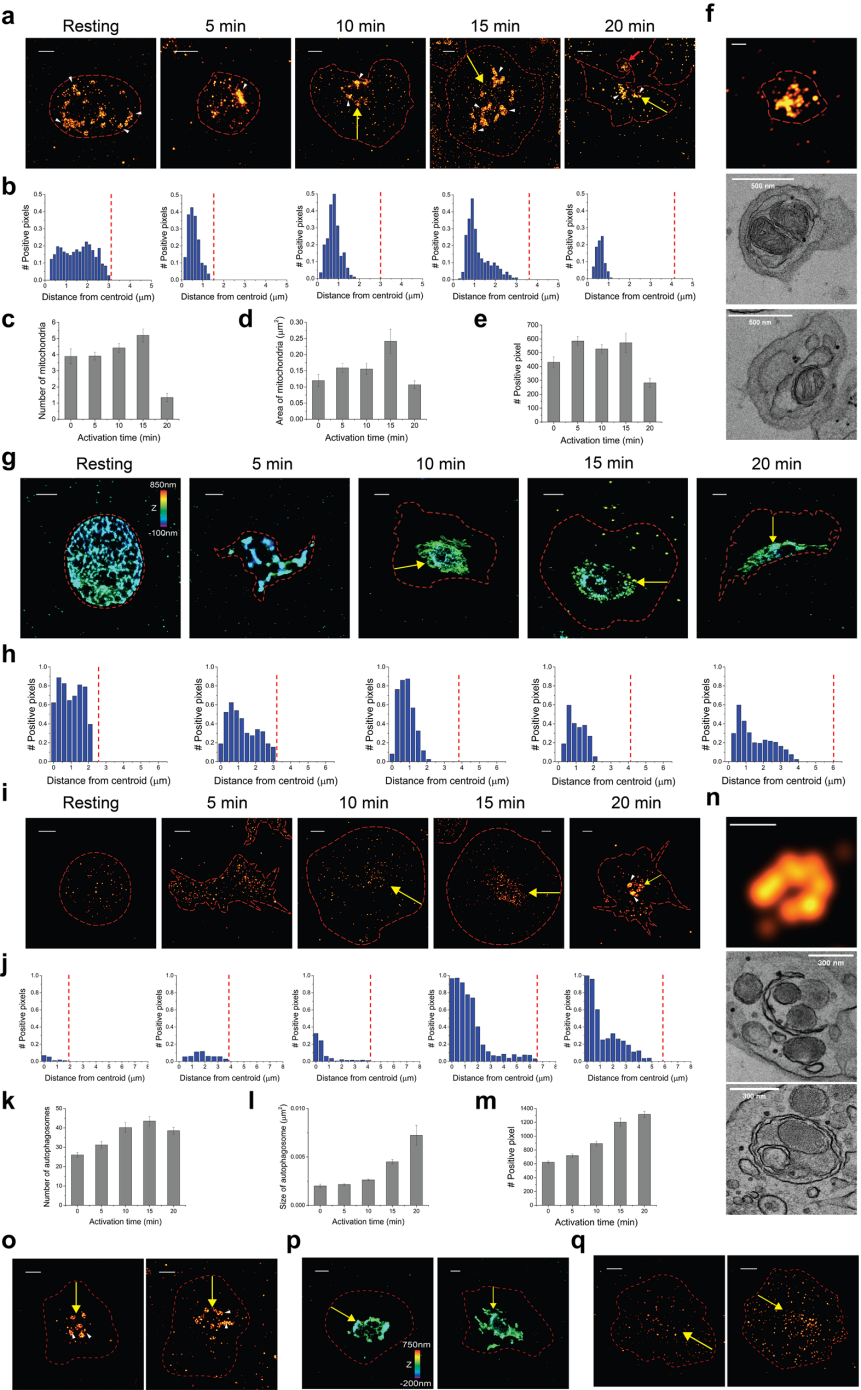
Super-resolution images of double membrane-bound organelles in activated platelets. (**a**) 2D STORM images of mitochondria in activated platelets. Mitochondria were observed from the platelets that were fixed at different activation time points (0, 5, 10, 15, and 20 min). Red arrow: released mitochondria from the activated platelet. Yellow arrows: centralization of mitochondria upon activation. (**b**) The radial distribution graph of mitochondria showing how mitochondria-positive pixel density varies as a function of distance from the centroid of the platelet is shown in (**a**), implying the centralization of mitochondria upon activation. (**c**–**e**) The bar graph indicates the number of mitochondria per platelet (**c**), the averaged area of mitochondria (**d**), and the averaged mitochondria-positive pixel number per platelet (**e**) upon activation induced by PMA (mean ± SD, n = 19). (**f**) Representative 2D STORM (top) and TEM (middle, bottom) images of released mitochondria encapsulated in microparticles. (**g**) 3D STORM images of DTS in activated platelets. DTS was observed from the platelets that were fixed at different activation time points (0, 5, 10, 15, and 20 min) (yellow arrows: centralization of DTS upon activation). (**h**) The radial distribution graph of DTS showing how DTS-positive pixel density varies as a function of distance from the centroid of the platelet is shown in (**g**), implying the centralization of DTS upon activation. The vacant space in the center was observed at 10–15 min, probably suggesting OCS regions. (**i**) 2D STORM images of autophagosomes in activated platelets. Autophagosomes were observed from the platelets that were fixed at different activation time points (0, 5, 10, 15, and 20 min) (yellow arrows: centralization of autophagosomes upon activation). (**j**) The radial distribution graph of autophagosome showing how autophagosome-positive pixel density varies as a function of distance from the centroid of the platelet shown in (**i**), implying the centralization of autophagosome upon activation. (**k**–**m**) The bar graph indicates the number of autophagosome per platelet (**k**), the averaged area of autophagosome (**l**), and the averaged autophagosome-positive pixel number per platelet (**m**) upon activation induced by PMA (mean ± SD, n = 18). (**n**) Representative 2D STORM (top) and TEM (middle, bottom) images of cup-like (middle) and shell-like (bottom) structure of autophagosomes. (**o**–**q**) STORM images of mitochondria (**o**), DTS (**p**), and autophagosome (**q**) in nocodazole-pretreated activated platelets, implying the centralization of organelles (yellow arrows) upon activation even in nocodazole treatment. Scale bar 1 μm in (**a**,**g**,**i**,**o**,**p**,**q**), 500 nm in (**f**) and 300 nm in (**n**).

We next examined the ultrastructural changes of double membrane-bound organelles during the platelet activation process, including mitochondria, DTS, and the autophagosome. From the STORM images of mitochondria, we observed an average of seven mitochondria per resting platelet, which is consistent with previous reports^29^ (Fig. 4a, Supplementary Fig. S7a). To quantify such distribution changes, we calculated the radial density distribution of localization derived from the 2D STORM images by determining the radial distance of each localization relative to the centroid of a platelet. These homogeneously distributed mitochondria were transported to the middle-central part of the activated platelets with an increase in their size (Fig. 4b). This quantification showed that the density of the mitochondria decreased in 20 min, probably due to the release of mitochondria through microparticles. Microparticles derived from platelets play a role in thrombus formation and blood clotting^30^ (Fig. 4c–e). We observed the released mitochondria encapsulated in microparticles from STORM images and TEM images (Fig. 4f). Mitochondria have been suggested to be critical in platelet survival, thus determining platelet lifespan^31^. It is also known that these extracellular mitochondria could lead to inflammatory responses^32^. Therefore, increased mitochondria and their release are likely essential steps for platelet activation, followed by inflammation development.

Next, we performed STORM imaging of DTS in resting and activated platelets. Although platelets do not have a ribosomal endoplasmic reticulum (ER), they have a smooth endomembrane system that originates from the ER, which is called DTS^29^. It is known to store calcium for cell activation^10^. It has also been reported to exhibit an entangled structure due to a high degree of intertwining with the OCS, as their close proximity may serve to transfer extracellular signals to DTS for the local release of calcium^29^. We also observed such an entangled structure of the DTS from our super-resolution image data, and this has not been resolved using conventional fluorescence microscopy (Fig. 4g, Supplementary Fig. S7b). The homogeneously distributed intertwined DTS in a resting platelet was centralized with a small number of vacant small spaces in the middle of a spread platelet upon activation, and this is likely to be closely associated with the OCS (Fig. 4h). Notably, we could not observe any DTS signal outside the ‘yolk’ region in the ‘fried-egg’ morphology in the spreading activated platelets, regardless of the type of ER marker (Supplementary Fig. S8). This could be because there is not enough space in this area for DTS to present, because we observed a significantly narrow space in this area from the x–z cross-section of β2-spectrin and vimentin.

Lastly, we performed STORM imaging of the autophagosome in activated platelets using LC3B antibody as an autophagy marker (Fig. 4i, Supplementary Fig. S7c). The STORM images of autophagosomes revealed relatively smaller-sized puncta compared to other organelles, but showed the similar centralized localizations upon activation (Fig. 4j). By quantifying the LC3B-positive pixel number per platelet as the quantity of autophagosomes during the activation process, the increased quantity of autophagosome was observed upon activation by PMA (Fig. 4k–m). Although Feng et al*.* reported that the autophagosome of platelets was independent of platelet activation, recent studies have proposed that autophagy is induced upon platelet activation^33,34^. Related to these recent studies, we observed the cup-like structure of the autophagosome cluster from the STORM image at the later activation time point (20 min) (Fig. 4n). Interestingly, this cup-like or shell-like structure of autophagosomes was also observed in TEM images of activated platelets, which have not been reported before, to the best of our knowledge. Although the autophagosome is important for maintaining cellular homeostasis, questions remain regarding the subcellular ultrastructure and location of autophagosomes in activated platelets. Our findings not only demonstrate the resolved well defined structures of autophagosomes but also the granules sequestered by autophagosomes in activated platelets. Collectively, our data demonstrate that the autophagosomes of platelets mostly do not show significant changes during platelet activation, but show the cup-like or shell-like structure of autophagosomes in the last stages of activation.

Our findings on the centralization of double membrane-bound organelles within activated platelets also raise important questions regarding the role of the cytoskeleton in platelet activation. To examine these roles, we investigated the effect of nocodazole pretreatments on organelle distribution of activated platelets, which disrupt the microtubules (Fig. 4o–q). It is difficult to examine the effect of cytochalasin D on organelle distribution of activated platelets, since the cytochalasin-D-pretreated activated platelets were not spread out; thus, we could not investigate the density distribution of organelles within this small area of non-spread platelets, as shown in Supplementary Fig. S4. Meanwhile, when the nocodazole-pretreated platelets were activated, we could still observe the centralization of organelles, including mitochondria, DTS, and autophagosome, which implies that their transportation to the center of the spread platelet is not guided by microtubules.

### Super-resolution imaging of secretory vesicles in activated platelets

**Figure 5:**
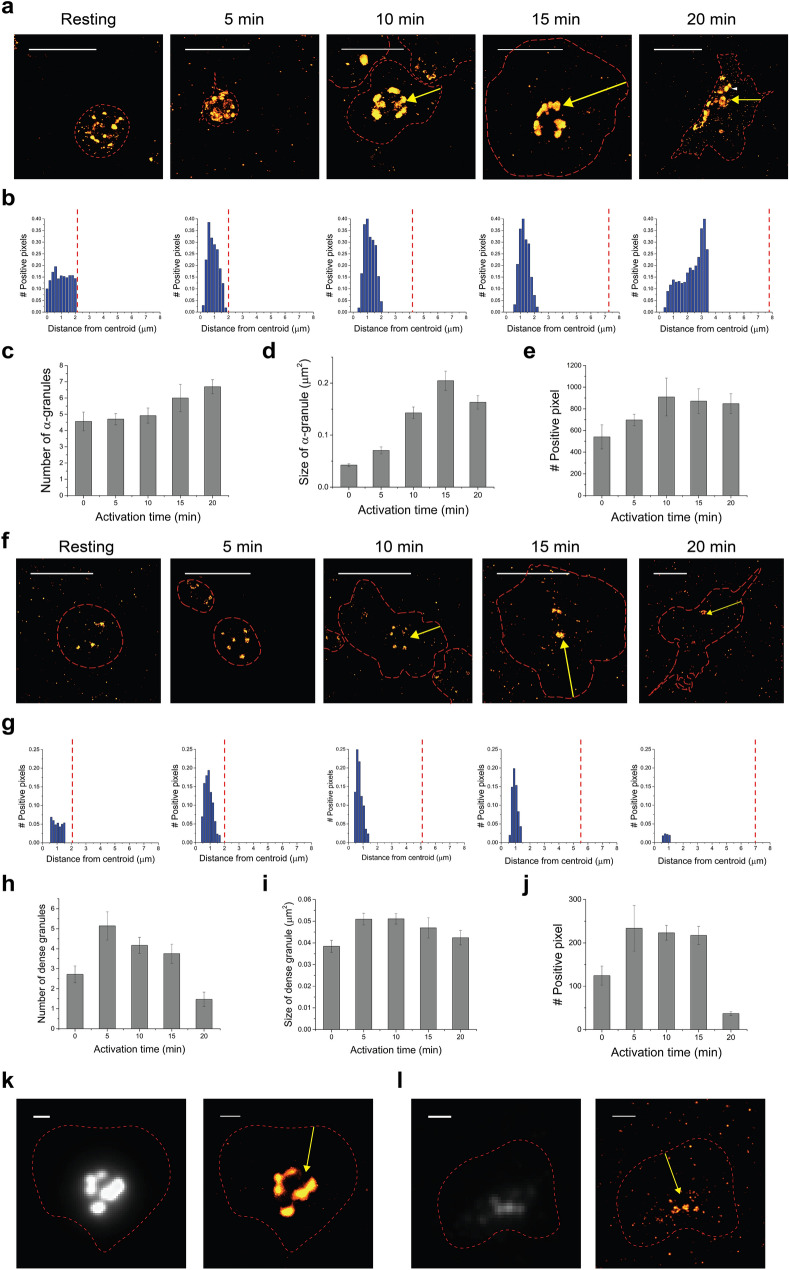
Super-resolution images of secretory vesicles in activated platelets. (**a**) 2D STORM images of α-granules in the activated platelet. α-granules were observed from the platelets that were fixed at different activation time points (0, 5, 10, 15, and 20 min) (yellow arrows: centralization of α-granules upon activation). (**b**) The radial distribution graph of α-granules showing how α-granules-positive pixel density varies as a function of distance from the centroid of the platelet is shown in (**a**), implying the centralization of α-granules upon activation. The vacant space in the center was observed at 10–15 min, probably suggesting OCS regions. (**c**) The bar graph indicates the number of α-granules per platelet upon activation induced by PMA (mean ± SD; n = 10–23). (**d**) The bar graph indicates the size of α-granules upon activation induced by PMA (mean ± SD; n = 19–28). (**e**) The bar graph indicates the averaged α-granule-positive pixel number per platelet upon activation induced by PMA (mean ± SD; n = 21). (**f**) 2D STORM images of dense granules in the activated platelet. Dense granules were observed from the platelets that were fixed at different activation time points (0, 5, 10, 15, and 20 min) (yellow arrows: centralization of dense granules upon activation). (**g**) The radial distribution graph of dense granules showing how dense granules-positive pixel density varies as a function of distance from the centroid of the platelet shown in (**f**), implying the centralization of dense granules upon activation. The vacant space in the center was observed at 10–15 min, probably implying OCS regions. (**h**) The bar graph indicates the number of dense granules per platelet upon activation induced by PMA (mean ± SD; n = 7–15). (**i**) The bar graph shows the size of dense granules per platelet upon activation induced by PMA (mean ± SD; n = 15–34). (**j**) The bar graph indicates the averaged dense granule-positive pixel number per platelet upon activation induced by PMA (mean ± SD; n = 19). (**k**,**l**) Conventional (left) and 2D STORM (right) images of α-granules (**k**) and dense granule (**l**) in nocodazole-pretreated activated platelets, implying the centralization of secretory vesicles upon activation (yellow arrows) even in nocodazole treatment. The red dashed line represents the boundary of the platelet identified from the corresponding DIC images. Scale bar 5 μm in (**a**,**f**) and 1 μm in (**k**,**l**).

In the last stage of platelet activation, the activated platelet needs to recruit other platelets to form a platelet aggregate as a hemostatic plug. To employ additional platelets, activated platelets secrete the contents of platelet granules through the OCS^29^. The platelet granules contain hundreds of proteins and they are the primary storage and secretory organelles of platelets; hence, their deficiencies are known to lead to a variety of bleeding disorders by impairing clotting response^35^. To investigate the associated ultrastructural changes of platelet granules during the activation process, we performed STORM imaging of α-granules and dense granules and analyzed their distribution in resting and activated platelets (Fig. 5). First, we used thrombospondin-1 antibody for α-granule labeling, because it is known to be a major constituent of α-granules^36^. The α-granules in the platelets from the STORM images revealed a considerable morphology and size heterogeneity, as previously observed (Fig. 5a,b, Supplementary Fig. S9a)^10^. We observed that the average size of α-granule clusters increased until 10 min without significant changes in number, followed by a slight decrease in their size in the later stages of the activation process (Fig. 5c,d). This increased quantity of α-granules at the beginning of the activation process is consistent with previously reported increased expression level of α-granules upon PMA stimulation (Fig. 5e)^23^. We also observed the centralized localization of α-granule clusters in the activated platelets in a pattern similar to that present in other organelles, such as mitochondria, DTS, and autophagosomes (Fig. 5b). In a similar fashion to DTS, a few vacant small spaces were observed between the α-granule clusters localized in the central region of the activated platelets, which could probably be the space occupied by the closely located OCS. The close proximity between granules and OCS expedites the release of granule contents, and the observation of the decreased size of α-granule clusters in the later stages of activation could imply this release process.

Next, we also carried out STORM imaging for dense granules and their distribution quantification in the resting and activated platelets by using a serotonin antibody because it has been known that the dense granules store extremely high concentrations of serotonin (Fig. 5f,g, Supplementary Fig. S9b)^37,38^. In contrast to the α-granule cluster, a smaller size and number of dense granules per platelet with low morphology heterogeneity were observed in our STORM images. Most of them have a spherical shape with a mean diameter of 200–250 nm, which is similar to the value observed in previous studies. In a similar fashion to the α-granules, their quantity in terms of size and number increases at the beginning of activation, followed by a decrease in their quantity (Fig. 5h–j). They also showed a similar distribution pattern to the α-granules regarding a centralized localization upon activation. This centralization of granules has been reported to be induced by MB contraction; however, we could not observe any significant differences in their centralized localization from the nocodazole-pretreated activated platelets, as shown in Fig. 5k,l^39^. Therefore, the formation of a small ring of microtubules in the activated platelets is not likely to be the main force of the centralization of α-granules in the activated platelets. The role of actin in the centralization of granules could not be fully understood from the cytochalasin D treatment experiments, as it was difficult to notice their centralization from the non-spread platelets under the cytochalsin D treatment (Supplementary Fig. S10). Collectively, our results demonstrate that the quantity of granules increases at the beginning of the activation, and then they are centralized, probably since they are localized close to OCS for the secretion of their granular constituents and aggregation with other platelets.

### Super-resolution imaging of OCS in activated platelets

**Figure 6:**
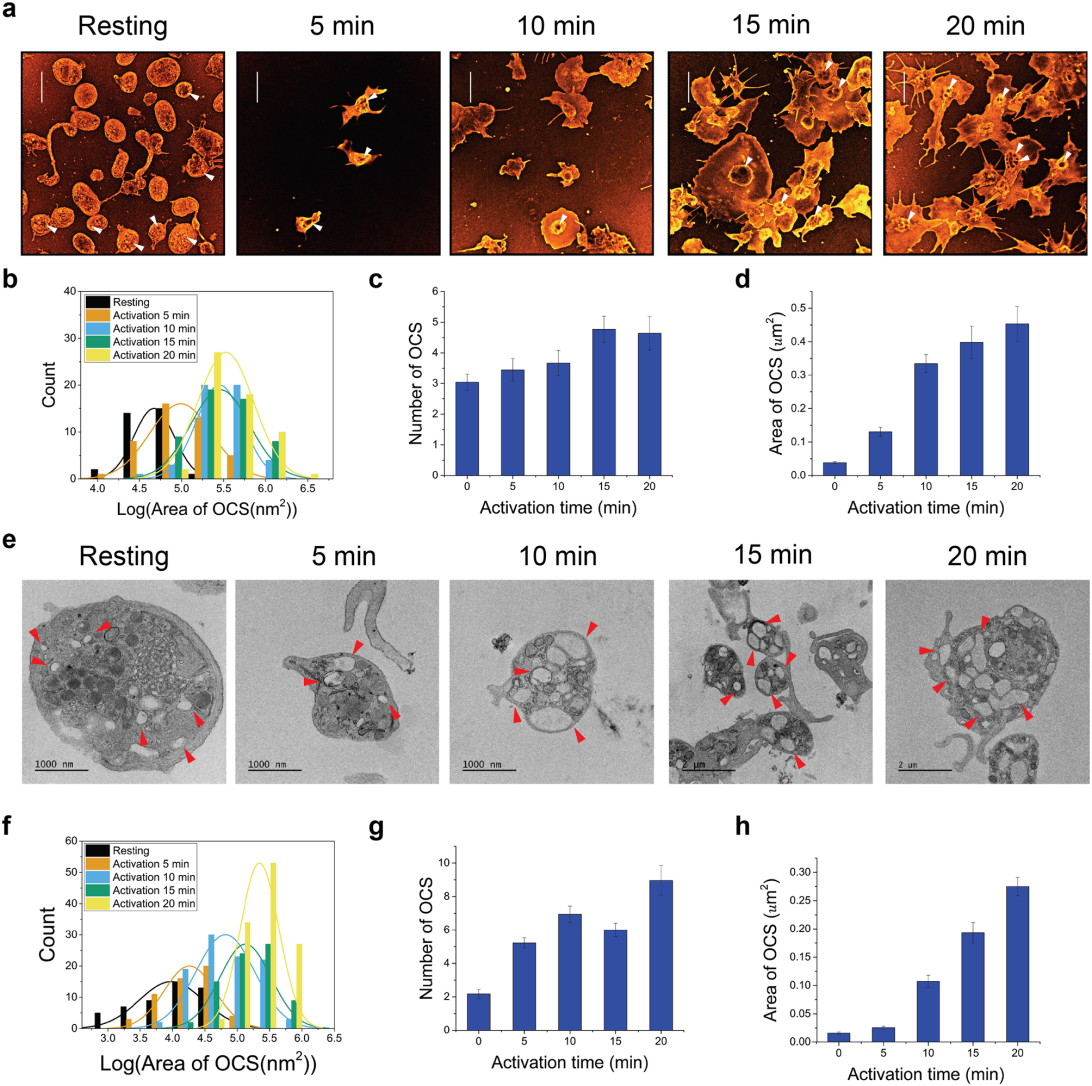
Super-resolution images of OCS in activated platelets. (**a**) 2D STORM images of a Nile Red-labeled activated platelet showing OCS. They were observed from the platelets that were fixed at different activation time points (0, 5, 10, 15, and 20 min) (white arrows: OCS). (**b**) The size distribution of OCS at different activation time points from 2D STORM images (n = 10). (**c**) The bar graph indicates the number of OCS per platelet upon activation induced by PMA from 2D STORM images (mean ± SD; n = 10). (**d**) The bar graph indicates the size of OCS per platelet upon activation induced by PMA from 2D STORM images (mean ± SD; n = 25–29). (**e**) TEM images of platelet were observed from the differentially-fixed samples at different activation time points (0, 5, 10, 15, and 20 min). Red arrows: OCS. (**f**) The size distribution of OCS at different activation time points from TEM images. (**g**) The bar graph indicates the number of OCS per platelet upon activation induced by PMA from TEM images (mean ± SD; n = 15). (**h**) The bar graph indicates the size of OCS per platelet upon activation induced by PMA from TEM images (mean ± SD; n = 18–34). Scale bar 5 μm in (**a**).

The OCS is a platelet intracellular part which consists of vacuolar structures with tubular neck regions and serves as a membrane reservoir during the spreading^10^. Thus, its structure is expected to change significantly upon activation. The OCS was mainly observed on the TEM image of the embedded and sectioned sample. However, the sectioned sample could not show the complete view of the large volume of OCS, limiting the interpretation of its three-dimensional structure in the entire activated platelet volume. To overcome this limitation, we used Nile Red as a membrane lipid stain. This method was successful in showing not only the mitochondria when permeabilized but also the ultrastructure of OCS, thus allowing us to quantify their number and size distribution at different activation time points (Fig. 6a–d, Supplementary Fig. S11–S13). As expected, the number and size of OCS increase upon activation, which implies an known increased need for OCS to release cargo in the activation process^29^. Notably, the OCS in the activated platelets is mainly localized in the center of the platelet. This is consistent with our aforementioned expectation, which suggests that the vacant spaces around the centralized organelles (i.e., mitochondria, DTS, autophagy, and granules) are occupied by the OCS. This suggestion was also examined using TEM images of the activated platelets, as shown in Fig. 6e. At the later stages of the activation, most of the area in the ‘yolk’ region of the ‘fried-egg’ shaped spread platelets were occupied by an enlarged OCS, and they were surrounded by other organelles, such as mitochondria, DTS, autophagy, and granules. There was an increase in the number and size of the OCS upon activation, as observed in TEM (Fig. 6f–h and STORM images (Fig. 6b–d).

**Figure 7:**
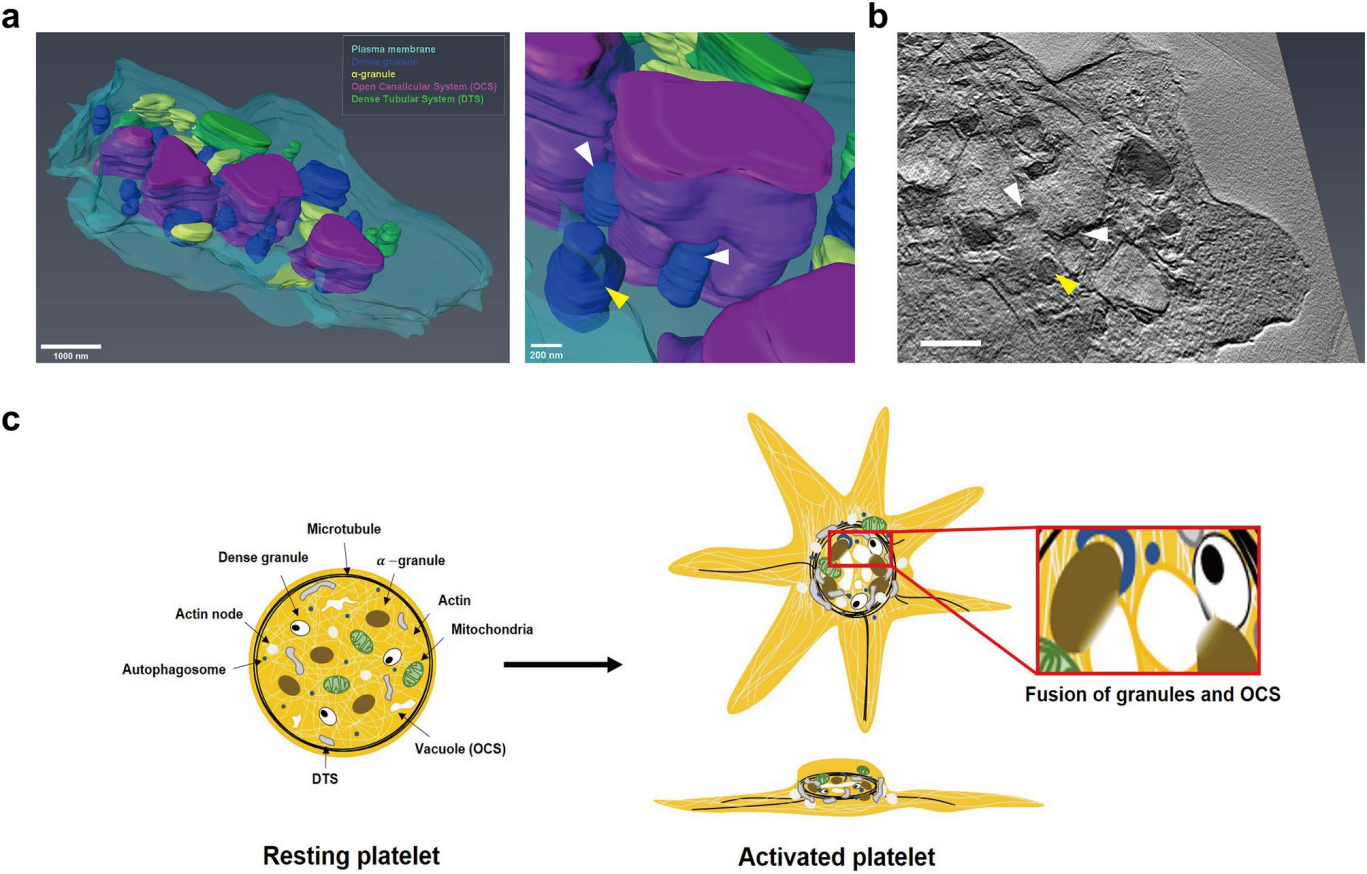
The structural organization of activated platelets. (**a**) The 3D reconstructed HV-EM image of activated platelets visualized at different angles. The fusion of granules and OCS were observed (white arrows). (**b**) The cross-section images showing the fusion of granules and OCS (white arrows). Different membrane contrasts were observed in the fused granules (white arrows) and unfused granules (yellow arrow). Scale bar 1 μm. (**c**) Scheme showing the structural organization of resting and activated platelets.

Lastly, to observe this orchestration framework of various organelles around the OCS in the activated platelet, we carried out HV-EM imaging of the 700-nm-thin sections of the central region of activated platelets (Fig. 7a, Supplementary Video 3). Based on the different electron contrast from different organelles, we could identify the OCS, DTS, α-granule, and dense granule. From the reconstructed three-dimensional electron tomogram, we observed that α-and dense granules are closely surrounding the OCS, and many of them are in contact with the OCS by showing the decreased membrane contrast, which probably implies the formation of routes of cargo release (Fig. 7b). In contrast to the granules, the DTS revealed no physical conjunction with the OCS, even when they were close to each other. Therefore, we demonstrated the close association of the granules and the OCS in the activated platelets by using the HV-EM three-dimensional tomogram (Fig. 7a,b).

## Discussion

By combining STORM imaging, live-cell holographic tomography, DIC microscopy, various EM techniques, correlative STORM and EM, and their quantitative cluster analysis, we have resolved the native ultrastructure of activated platelets induced by PMA, which have not been well investigated, compared to other agonists. This integrating investigation enabled quantitative and systematic examinations of the structural organization and relative arrangement at the nanoscale. Although some of the imaged organelles exhibited punctate and speckled patterns, we confirmed that these patterns are not derived from the non-specific binding of antibodies (Supplementary Fig. S14).

First, we showed that the actin mesh network within a resting platelet was converted into actin bundles with the formation of actin nodules upon activation, and the activated platelets were spread out from the center of actin nodules along the direction of actin bundles. Our finding that actin-disrupted activated platelets could not be spread out at all suggests that actin organizations, especially actin nodules and bundles, provide the main force for the spreading and determine the overall shape of platelets, including the direction and origin of spreading. We also showed that pseudopodia mainly consist of actin, and their formation seems to be required to probe the environment and recruit other platelets for aggregation during activation; hence, actin plays an important role in the activation process. The conversion from MB to the formation of small microtubule rings observed during the activation process matches well with previous studies, and we further examined the full unwinding and depolymerization processes of microtubules in the middle of the conversion in the central regime of spread platelets^29^. Interestingly, we observed that the microtubules in the small microtubular ring are heavily acetylated, while the depolymerized microtubules outside of the small ring in the activated platelets are not acetylated. We also found that the actin nodules were not centralized with a slightly reduced number in the microtubule-disrupted activated platelets, compared to the control sample, which raises the possibility that microtubules can play a role in the formation of actin nodules during the activation process. We also observed the contractility of activated platelets under nocodazole treatment. As the GEF-H1/RhoA/ROCK signaling pathway is known to play a critical role in mediating cell contractility, it is likely that nocodazole-induced microtubule depolymerization in activated platelets results in RhoA activation by the released GEF-H1 from microtubules that may be followed by the contractility of activated platelets^40^. Other cytoskeletal elements including βII-spectrin and vimentin, revealed the centralization of their localization upon activation, which area is observed to cover the centralized area of microtubule small ring, mitochondria, α-granules, and dense granules from two-color STORM images (Supplementary Fig. S15). Although the role of these cytoskeletal elements during platelet activation has not been fully understood yet, we suggest that they could be crucial for stabilizing the extended structure of the central bump region because of the densely centralized organelles in the activated platelets. Collectively, the activated platelets were observed to exhibit different morphology, flexibility, and spreading effects depending on their activation states, and these states can be determined by several factors related to the cytoskeletal elements, including the formation of actin bundles and nodules, the flexibility of microtubules, the effect of microtubules on the actin nodule formation, and the centralized βII-spectrin and vimentin during the activation process.

Our work further demonstrates the centralization of double membrane-bound organelles during the platelet activation process, including mitochondria, DTS, and autophagosome, suggestive of distinct zones of organelle packaging within activated platelets (Fig. 7c). Their centralizations could be due to either the limited space of the peripheral thin regions or the close association with the OCS, which is also localized in the center of activated platelets. These centralized distributions of OCS, mitochondria, and DTS in activated platelets have not been known so far since previous studies assumed in their models that they are homogeneously distributed across the whole volume of activated platelets, only based on their observation from the TEM images of ultrathin sections^39^. Moreover, our results revealed a cup-like or shell-like scaffold of autophagosomes in activated platelets, which has not been reported before, to the best of our knowledge.

Similar to the double membrane-bound organelles, we also showed that granules were concentrated in the center of the activated platelets. Although this centralization could be induced by the contraction of the MB, as previously reported, our results demonstrate that microtubule-disrupted activated platelets still show granule centralization, which suggests that microtubules are not the main driving force to transport granules to the center of spread platelets^39^. Instead of microtubules, we suggest that their centralizations could be due to either the limited space at the peripheral thin regions or the close association with the OCS that was also observed to be localized in the center of activated platelets.

Finally, quantitative analysis showed that the expression levels of both α-granules and dense granules increased until the middle stage of the activation process and then slightly decreased in the later stage, and this has not been previously quantified at the single platelet level. This release of the contents in the granules could be through the closely associated OCS or the autophagy process, as observed. Remarkably, we observed that platelet granules fuse with the OCS to release their cargo from HV-EM images, which has not been resolved in previous studies, to the best of our knowledge. This fusion between granules and the OCS could trigger granule secretion for the recruitment of additional platelets, resulting in an enlargement of the OCS with an increase of surface area and sequestration of proteins, as suggested in previous studies^29^.

Our findings highlight how recently developed super-resolution microscopy can help elucidate the molecular mechanisms of the platelet activation process, which has not been investigated at the nanoscale before. We anticipate that future live-cell multi-color super-resolution microscopy experiments may directly visualize these intermolecular processes within activated platelets in real-time.

## Methods

### Cell culture and drug treatment

This study was approved by the Institutional Review Board (IRB) of Severance Hospital (Seoul, Republic of Korea), an affiliated hospital of Yonsei University Health System (Approval Number: 4-2012-0336). All participants provided written informed consent before the donation of blood samples. Blood samples were acquired with the approval of the IRB and all experiments were performed in accordance with relevant guidelines and regulations. Whole blood from healthy adult volunteers was drawn into sodium citrate tubes and centrifuged (250×*g*) for 15 min at room temperature. The supernatant, which was platelet-rich plasma (PRP), was diluted in Dulbecco’s phosphate buffered saline (DPBS), and then plated on a glass-bottom confocal dish at 37 °C and 5% CO_2_ for 1 day before fixation and activation. We did not coat any chemical reagents onto the glass to avoid any possible effects on platelet activation. In contrast, many previous platelet studies have used poly-lysine or bovine serum albumin to decrease the possible charging effect from glass coverslips. To assess if the bare glass induced platelet activation in our experiments, we tested the glass surface effects on platelet morphology using poly-L-Lysine. The PRPs plated on the poly-L-lysine-coated glass-bottom confocal dish do not show any significant differences in morphology, thus implying that the surface of bare glasses does not significantly affect to the PRP morphology (Supplementary Fig. S16). Although we did not see significant charging effects of glass surface on the platelet activation in our experiments, we expect that the use of albumin could be helpful to minimize surface-induced platelet activation, as previously reported^41^. For the activation, 0.1 µg/mL phorbol 12-myristate 13-acetate (PMA) (P1585; Sigma-Aldrich) was added at 37 °C and 5% CO_2_. For the disruption of microtubules and actin, 5 µg/mL nocodazole (M1404; Sigma-Aldrich) and 10$\upmu$ M cytochalasin D (C8273; Sigma-Aldrich) were added before the PMA treatment at 37 °C and 5% CO_2_ for 15 min, respectively.

### Imaging of live platelets

For the DIC and fluorescence imaging of live platelets, the PRP were cultured and activated in the same way, and the DIC and fluorescence images were obtained at 10 Hz. For fluorescence imaging of actin, we stained the samples with 1 μM SiR-actin (CY-SC006; Cytoskeleton) with 10 μM verapamil for 1 h at 37 °C and 5% CO_2_.

For the 3D tomogram of live platelets, PRP diluted in DPBS was plated on a microscopic dish (TomoDish, Tomocube Inc.) at 37 °C and 5% CO_2_, and then covered with another glass coverslip (24 × 50 mm) for imaging. The 3D holotomographic imaging technique was performed on a HT-2H microscope (Tomocube Inc.), which reconstructs the 3D refractive index (RI) tomogram of a sample from multiple 2D holographic images of the sample obtained at various illumination angles. The 3D RI maps were rendered using commercial software (TomoStudio, Tomocube Inc.).

### Sample preparation for fluorescence imaging

For immunofluorescence, PRP was fixed in 3% (v/v) PFA (15714; Electron Microscopy Sciences) and 0.1% (v/v) GA (16020; Electron Microscopy Sciences) in DPBS for 15 min at RT. After washing three times with DPBS, samples were incubated with freshly prepared 0.1% (w/v) sodium borohydride (71320; Sigma-Aldrich) in DPBS for 6-8 min, permeabilized in 0.3% Triton X-100 in DPBS, and incubated in blocking buffer (3% [w/v] bovine serum albumin (BSA) in DPBS) for 30 min. Next, samples were stained with primary antibodies in blocking buffer for 30-60 min at RT; for example, anti-$\mathrm{\alpha }$-tubulin (ab6160; Abcam) was used for the microtubules, anti-$\mathrm{\beta II}$-spectrin (612562; BD Biosciences) for $\mathrm{\beta II}$-spectrin, anti-vimentin (AB5733; Merck Millipore) for vimentin, anti-thrombospondin 1 (MA5-13398; Invitrogen) for $\mathrm{\alpha }$-granule, anti-serotonin (ab6336; Abcam) for dense granules, anti-TOM20 (F-10) (sc-17764; Santa Cruz Biotechnology), and anti-TOM22 (ab246862; Abcam) for mitochondria, anti-LC3B (L7543; Sigma-Aldrich) for autophagy, and anti-tubulin acetylated antibody (T6793; Sigma-Aldrich) for acetylated tubulin. For DTS, we used a well-known ER marker (KLC3) antibody KLC3 (ab180523; Abcam) to image the ER structure^42^. To assess the non-specific binding of primary antibodies, various isotype control antibodies that correspond to the species of primary antibodies used were used instead of corresponding primary antibodies. For example, we used rat IgG isotype antibody (14-4321-82; Invitrogen), rabbit IgG isotype antibody (02-6102; Invitrogen), and mouse IgG isotype antibodies (31903; Invitrogen). The samples were washed three times with DPBS and stained with dye-conjugated secondary antibodies in blocking buffer for 60 min at RT. To assess the non-specific binding of secondary antibodies, the samples were stained only with dye-conjugated secondary antibodies in blocking buffer for 60 min at RT without previous staining with primary antibodies. After incubation with the secondary antibodies, cells were washed three times and post-fixed with 2% (v/v) PFA and 0.05% GA in DPBS for 10 min. Then, samples were stored at 4 °C in a 20 mM sodium azide (50489; TCI) solution in DPBS or immediately imaged in an imaging buffer. The imaging buffer was supplemented with 100 mM mercaptoethylamine (MEA) (30070; Sigma-Aldrich), 5% glucose (w/v), and oxygen scavenging enzymes (0.5 mg/mL glucose oxidase [G2133; Sigma-Aldrich] and 38 µg/mL catalase [C3515; Sigma-Aldrich]) in PBS at pH 8.5.

For the actin filament phalloidin labeling, samples were initially fixed and extracted with 0.3% (v/v) GA and 1% (v/v) Triton X-100 in cytoskeleton buffer (CB, 10 mM MES (2-(N-morpholino)ethanesulfonic acid) buffer, 150 mM NaCl, 5 mM ethylene glycol tetraacetic acid (EGTA), 5 mM glucose, 5 mM MgCl_2_, adjusted with NaOH to a pH of 6.1) for 10 min at RT, and then post-fixed for 20 min using 2% (v/v) GA in CB. After washing twice with DPBS, samples were incubated with a freshly prepared 0.1% (w/v) sodium borohydride solution for 6–8 min. Next, samples were stained with Alexa Fluor 647 conjugated phalloidin (A22287; Invitrogen) in DPBS overnight at 4 °C, briefly washed once with DPBS, and then immediately imaged using the aforementioned imaging buffer.

For the membrane staining with Nile Red, samples were fixed in the aforementioned way, and then stained using a 100 nM Nile Red (415711000, Acros Organics) solution in DPBS for 20–30 min at RT. After two brief washes, samples were immediately imaged in the imaging buffer solution containing 100–200 µM ascorbic acid (PHR1008; Sigma-Aldrich).

To perform two-color conventional fluorescence imaging of the membrane and mitochondria, the samples were fixed in the same way and then stained with anti-TOM22 primary antibody from rabbit and Alexa Fluor 647 conjugated donkey anti-rabbit secondary antibody (A31573; Invitrogen) in blocking buffer for 30 min and 60 min, respectively. After three washes, the samples were further stained with a 100 nM Nile Red solution for 20–30 min at RT and immediately imaged.

### STORM imaging

All STORM imaging experiments were conducted on a custom-built inverted microscope (Ti2-U; Nikon) equipped with a 100× objective lens (CFI SR HP Apo TIRF; Nikon) and 1.49 NA oil immersion. A 561 nm (OBIS; Coherent), a 647 nm (OBIS; Coherent), and a 405 nm (OBIS; Coherent) lasers were introduced into the sample through the backport of the microscope body. The samples were imaged with continuous illumination at 110 mW 561 nm, 120 mW 647 nm, and 0.1–1 mW 405 nm for dye activation and excited via total internal reflection fluorescence (TIRF) illumination. Fluorescence emission was filtered using a bandpass emission filter (LF408/488/561/635-B; Semrock) and imaged onto an electron-multiplying charge-coupled device (EMCCD) camera (iXon Ultra 888; Andor) at a frame rate of 60–100 Hz. The focus was maintained during data acquisition via the CRISP Autofocus system (ASI), which detects a separate IR beam signal reflected from the sample–liquid interface through an objective lens. For the 3D STORM imaging, a cylindrical lens with a focal length of 500 mm (LJ1144RM-A; Thorlabs) was inserted into the detection path, so that elongated point-spread functions (PSF) of single molecules were created with the information of the x, y, and z positions of single molecules. For the two-color imaging, each channel was obtained using a beam splitter (T635ipxr, T556ipxr; CHROMA) and a filter (ET670/50, ET585/65; CHROMA).

In STORM image analysis, each point spread function in the STORM image was fitted to a Gaussian function to determine the centroid position coordinates of individual fluorophores, and the reconstructed STORM image from the collection of centroids was rendered after drift correction, as previously described^43^.

### Image quantification

The intracellular distribution graph of organelles was analyzed using a custom written Matlab code. STORM images were median-filtered and then filtered by intensity according to Otsu’s thresholding algorithm, as previously reported^43,44^. To identify the boundaries of each organelle, we used 8-point connectivity, and their boundaries were dilated by 1 pixel. To determine their relative positions within a platelet, we performed correlated STORM and DIC imaging, and the boundaries and centroids of the platelets were identified from the correlated DIC images. The Euclidean distances between particular organelle-containing pixels and the centroid of a platelet were determined and then used to determine the intracellular distribution of organelles. The localizations were then binned, and the density fraction was normalized to the area of each radial slice.

For the directionality analysis of the STORM images of actin, we used the plugin ‘directionality’ of ImageJ. Briefly, this plugin allows us to compute a histogram indicating the amount of structures in a given direction to infer the preferred orientation of structures from the input image. If the input images have a completely isotropic content, it provides a flat histogram. In contrast, if there is a preferred orientation in the input images, it gives a histogram with a peak in that orientation. The peak in a histogram was fitted using a Gaussian function, and the intensity of a Gaussian peak was calculated as the non-homogeneity of directionality.

### Sample preparation for EM

For SEM imaging, the plated PRP was briefly washed once with pre-warmed DPBS, initially fixed and extracted with 0.3% (v/v) GA and 1% (v/v) Triton X-100 in CB for 10 min at RT, followed by post-fixation for 20 min using 2% (v/v) GA in CB. Next, the samples were incubated with a 0.1% (w/v) tannic acid (403040; Sigma-Aldrich) solution in distilled water using a 25-mm syringe filter for 20 min at RT. After three washes with distilled water for 10 min, samples were incubated with filtered 0.1–0.2% (w/v) uranyl acetate (UA) in distilled water for 20 min at RT. After washing again with distilled water for 10 min, samples were dehydrated using a graded ethanol series, and further dehydrated with hexamethyldisilazane (HMDS) for 15 min. The samples were placed on a silicon wafer, followed by a Pt coating. The samples were imaged using an S-4800 Field Emission Scanning Electron Microscope (FESEM, Zeiss) at 3 keV.

For TEM imaging, the plated PRP was fixed with 2.5% GA in 0.1 M cacodylate buffer (pH 7.4) overnight at 4 °C. Following three washes with cacodylate buffer, the samples were post-fixed with 1–2% osmium tetroxide in 0.1 M cacodylate buffer on ice for 1 h and washed thrice with cacodylate buffer. After dehydration using a graded ethanol series, the samples were infiltrated using progressive incubations with propylene oxide and Epon 812 and embedded in 100% Epon 812 resin. Polymerization was conducted with 100% Epon 812 at 70 °C for 24 h. Ultrathin sections (~ 70 nm) were obtained using an EM UC7 ultramicrotome (Leica, Austria) and collected on 100–150 mesh copper grids. After staining with UA and lead citrate, the sections were visualized using the KBSI Bio-HVEM System [JEM-1400 Plus at 120 kV (JEOL, Japan)].

For HV-EM, the sample was prepared similarly, but 1–2% osmium tetroxide and 1.5% potassium ferrocyanide in 0.1 M cacodylate buffer were used for post-fixation to increase contrast, and relatively thick sections (~ 700 nm) were obtained to reconstruct the three-dimensional ultrastructure. HV-EM imaging was performed on the KBSI Bio-HVEM System [JEM-1000BEF at 1,000 kV (JEOL, Japan)]. The cell was tilted from + 50 to − 50° in increments of 2°. A total of 51 tilt images were recorded by TEM Recorder software (JEOL System Technology, Tokyo, Japan), and digitized tilting images were aligned and tomographically reconstructed using Composer and Visualizer-Kai software (TEMography.com, System In Frontiers Inc., Tokyo, Japan). Surface rendering and 3D modeling were performed using AMIRA software [Thermo Fisher Scientific (FEI), Hillsboro, OR].

### Sample preparation for correlative STORM and SEM

For correlative STORM and SEM imaging, the PRP plated on the coverglass was fixed with 0.3% (v/v) GA and 1% (v/v) Triton X-100 in CB, and then post-fixed with 2% (v/v) GA in CB. After immuno-labeling in the aforementioned way, the samples were imaged using STORM. After STORM imaging, the coverglass was disassembled from the attached confocal dish by removing the adhesive with ethanol. After washing three times with distilled water, the SEM sample preparations were performed as described above. The samples were imaged using SEM after identifying the same regions that were imaged using STORM through the linear transformation of reference coordinates detected both in the STORM and the corresponding SEM images. The STORM image and the corresponding EM image were overlaid by rescaling, translation, rotating, and adjusting the brightness and contrast of the EM images using Icy software based on the features visualized in both images as fiduciary markers.

## Supplementary Information

Supplementary Video 1.

Supplementary Video 2.

Supplementary Video 3.

Supplementary Information 1.

## Data Availability

The authors declare that the data supporting the findings of this study are available within the paper and its supplementary information files.
