# Supplementary material for: Super-resolution imaging of platelet-activation process and its quantitative analysis
Source: Sci Rep. 2021 May 18;11:10511. doi: 10.1038/s41598-021-89799-9 (PMC8131365; doi:10.1038/s41598-021-89799-9)
Supplement: Supplementary file 4 — Supplementary Information 1. [file 41598_2021_89799_MOESM4_ESM.pdf]

Super-resolution imaging of platelet-activation process and its quantitative analysis

Jinkyoung Chung[1], Dokyung Jeong[1], Geun-ho Kim[1], Seokran Go[1], Jaewoo Song[2], Eunyoung Moon[3], Yang Hoon Huh[3], Doory Kim[1,4,5,6*]

*Correspondence to doorykim@hanyang.ac.kr

Affiliations

[1]Department of Chemistry, Hanyang University, Seoul 04763, Republic of Korea

[2]Department of Laboratory Medicine, Yonsei University College of Medicine, Seoul 03722, Republic of Korea

[3]Electron Microscopy Research Center, Korea Basic Science Institute, Cheongju 28119, Republic of Korea

[4]Research Institute for Convergence of Basic Sciences, Hanyang University, Seoul 04763, Republic of Korea

[5]Institute of Nano Science and Technology, Hanyang University, Seoul 04763, Republic of Korea

[6]Research Institute for Natural Sciences, Hanyang University, Seoul 04763, Republic of Korea

Supplementary Note 1

Our observation suggests that platelets may initiate their activation processes upon contact with the coverglass, even without any agonist. However, when we waited for the further activation of platelets until five days of plating on the coverglass, we could not observe significant structural differences which could be considered an activation process. It implies that the platelets cannot further proceed with the activation process or that the activation process is too slow to be observed without the use of an agonist. Therefore, we used PMA to expedite the activation process for observation. (Supplementary Fig. S1)

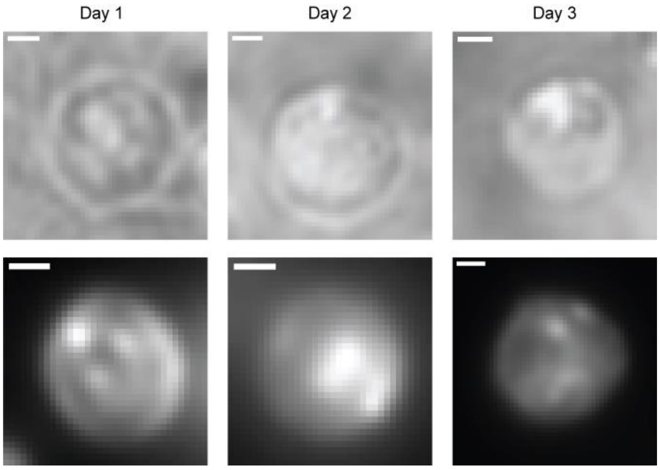

Figure S1.

The differential interference contrast (top) and fluorescence images (bottom) of platelets at different days, which are not activated by PMA, showing no significant morphological changes up to 3 days. For fluorescence imaging, the actin was stained by SiR-actin. Scale bar: 1 μm.

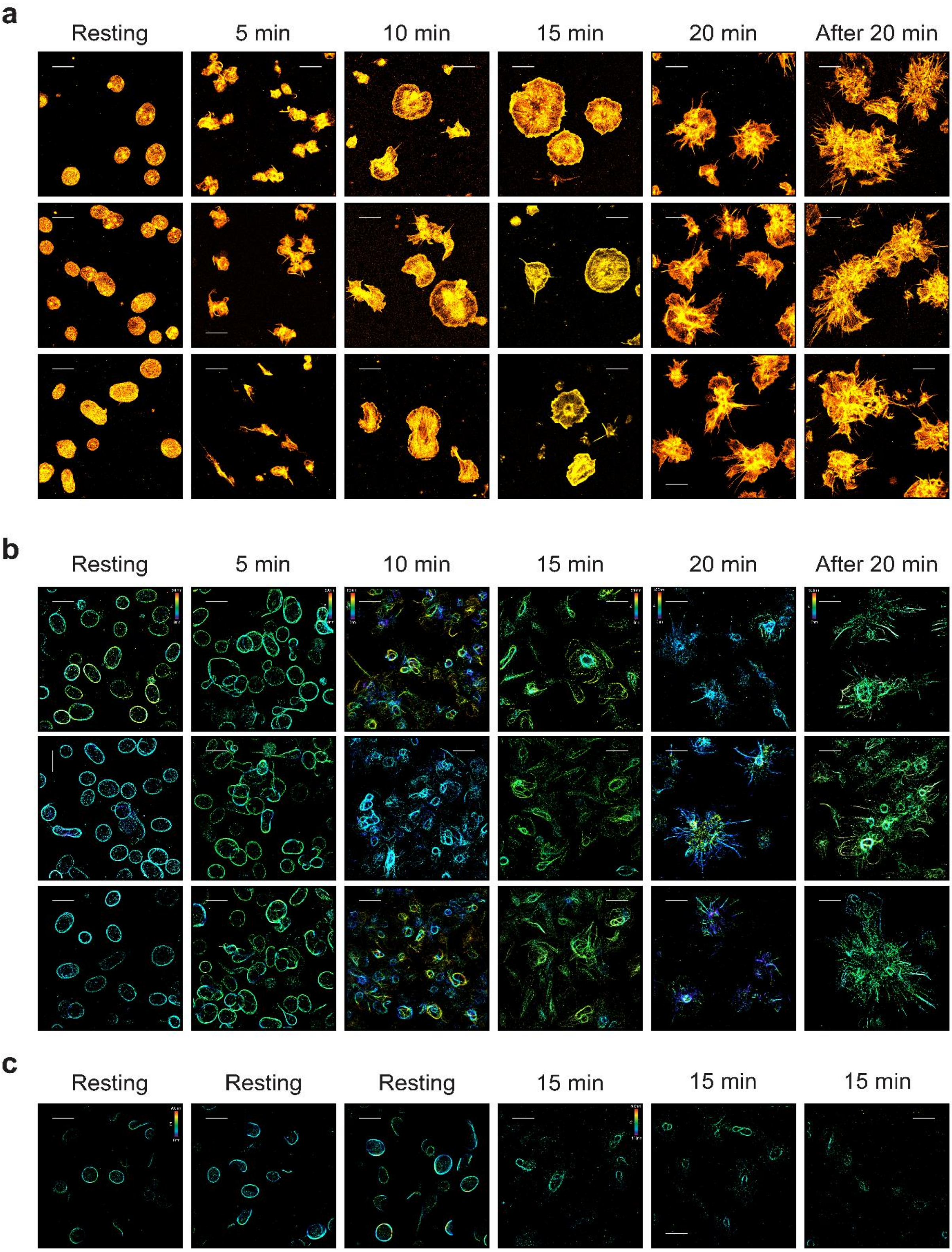

Figure S2.

(a) 2D STORM image examples of actin filaments in activated platelets. (b) 3D STORM image examples of microtubules in the activated platelet. (c) 3D STORM image examples of acetylated microtubules in the resting and the activated platelet. Scale bar: 5 μm.

Supplementary Note 2

Aggregation between the spread platelets was also observed at early time points, when they were nearly located (Supplementary Fig. S3). However, we tried to image the separated platelets at each activation time point to observe the resolved ultrastructure of individual activated platelets.

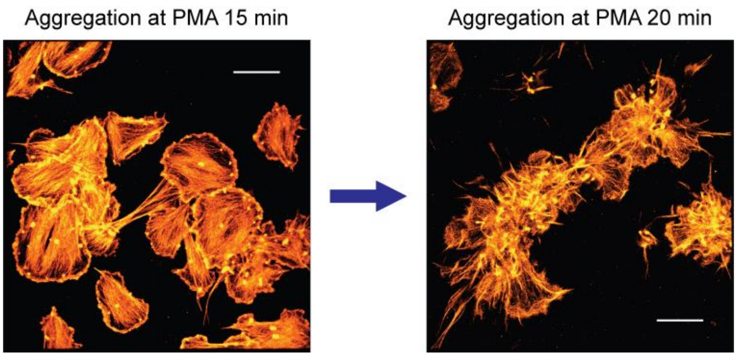

Figure S3.

STORM images of actin filaments in the aggregated platelets at different activation time points. Scale bar: 5 μm.

Supplementary Note 3

We also performed STORM imaging of acetylated microtubules, and found that these were mainly present in the small microtubular ring and not in the depolymerized microtubules outside the small ring (Fig. 2i).

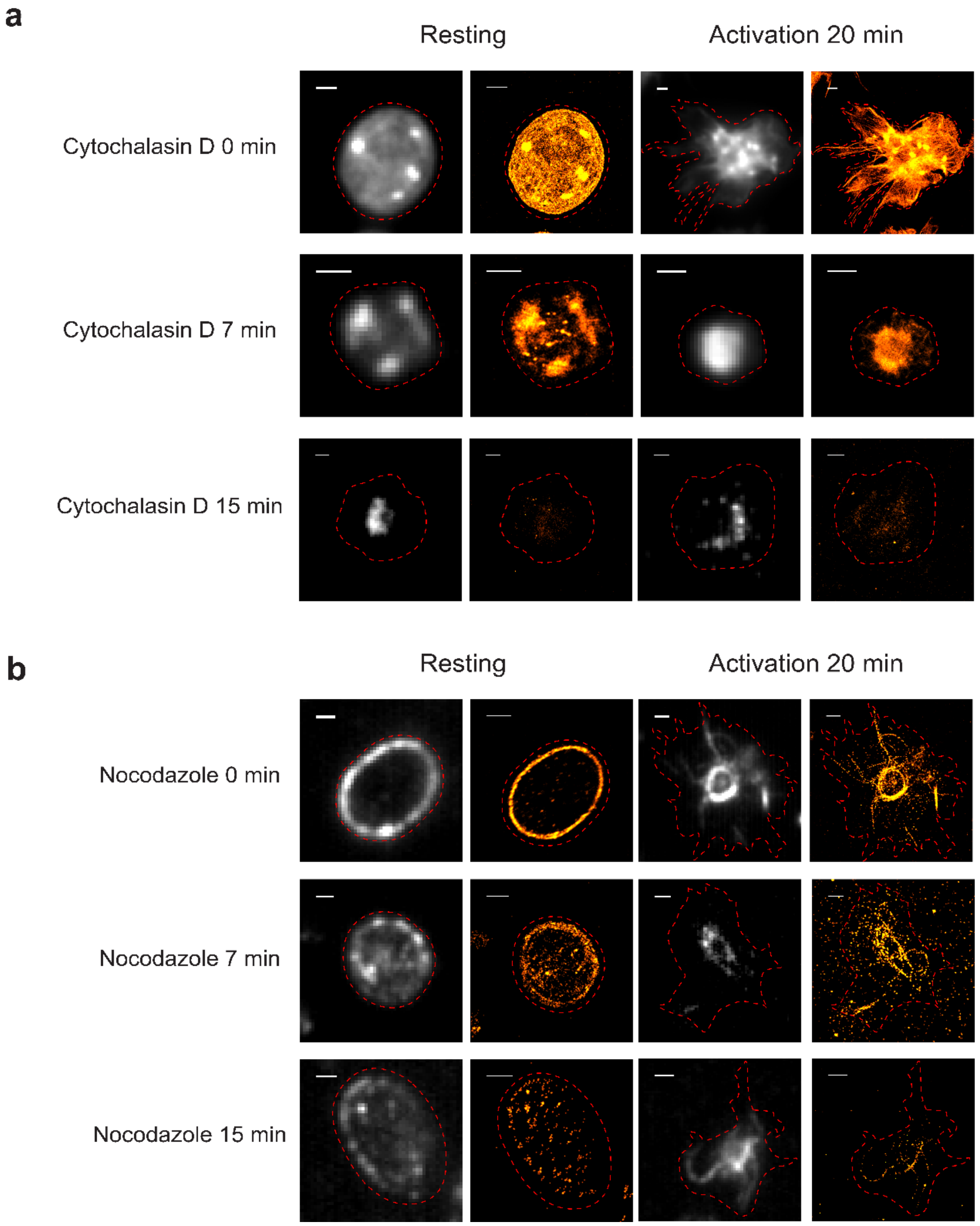

Figure S4.

Effect of cytochalasin D and nocodazole treatment on actin and microtubules in resting and activated platelets, respectively. Representative time-lapse diffraction-limited and STORM images of (a) actin and (b) microtubules in resting (left) and activated (right) platelets. (top) Prior to treatment (middle) 7 min after drug treatment, and (bottom) 15 min after drug treatment. Complete depolymerization of actin and microtubules was noted after 15 min of drug treatment. Scale bar: 1 µm.

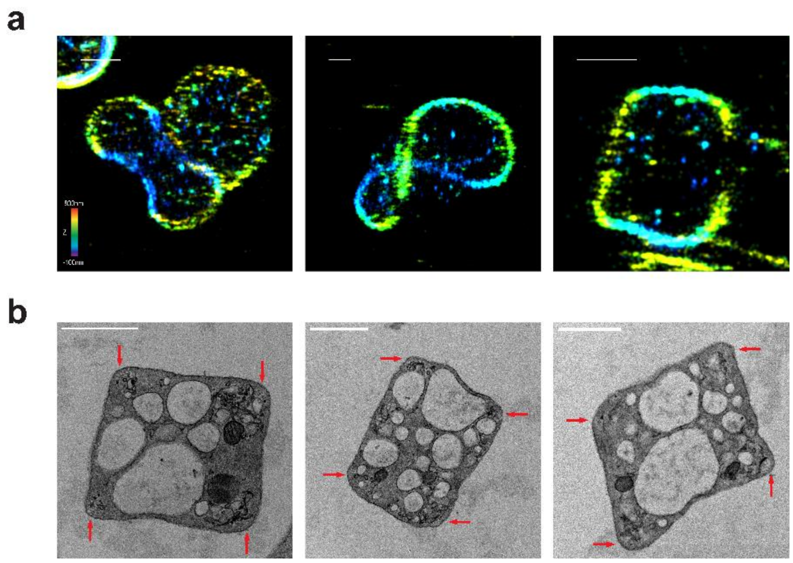

Figure S5.

(a) 3D STORM images of microtubules exhibiting the ‘potato chip’ like structure of microtubules in cytochalasin D-pretreated activated platelets to provide additional examples to those provided in Figure 3(d). (b) TEM images of the cytochalasin D-pretreated activated platelets showing the microtubule bundles at four corners of the ‘potato chip’-like structure of microtubules as other examples of the data presented in Figure 3(d). TEM images of the cytochalasin D-pretreated activated platelet showing the microtubule bundles at four corners of the ‘potato chip’-like structure of microtubules (red arrow). Scale bar: 1 μm.

Supplementary Note 4

We performed 3D STORM imaging to elucidate the ultrastructural changes of the other cytoskeletons during the activation process, including spectrin and vimentin, since their localization has not been well explored in super-resolution. To observe the spectrin, we used the βII-spectrin antibody since βII-spectrin is known to be abundantly expressed in platelets as the major spectrin isoform of nonerythroid cells[1]. From the STORM images of β2II-spectrin in a resting platelet, we observed their punctate and speckled pattern and uniform distribution, which were also previously observed from the STORM images of erythrocytes, thus suggesting that they were real structures and not random dots (Supplementary Fig. S6a). [2] Upon activation, they were concentrated in the center of the activated platelets at a relatively high position, probably localized in the ‘yolk’ spot of ‘fried egg’ morphology of activated platelets. Since the spectrin-based membrane skeleton is known to line the inner plasma

membrane, the hollow shape in the swollen central region of the activated platelet was observed from the x-z cross-section of the 3D STORM images. Their increased density in the center was slightly decreased at the later stages of activation (~20 min), which could be due to the released microparticles encapsulated by spectrin.

We also performed 3D STORM imaging for vimentin intermediate filaments. We observed that they exhibit a punctate localization in platelets, as previously observed (Supplementary Fig. S6c)[3]. It is known that the vimentin network is exposed on the surface of activated platelets, and we could also observe that it is bound to the surface of the activated platelets from the x-z cross-section of 3D STORM images[3]. Moreover, the higher level of vimentin at 5 min compared to levels in the resting platelet could be possibly due to the protein synthesis induced by various needs within the platelet as previously suggested [4]. We also found that they are centralized in the 'yolk' region of 'fried-egg' morphology with increased density during the activation process, in a similar fashion to βII-spectrin. (Supplementary Fig. S6d) We also observed a high density of vimentin on the released platelet microparticles from the activated platelets, probably wrapping the proteins released from the α-granules or organelles such as mitochondria, as previously suspected.

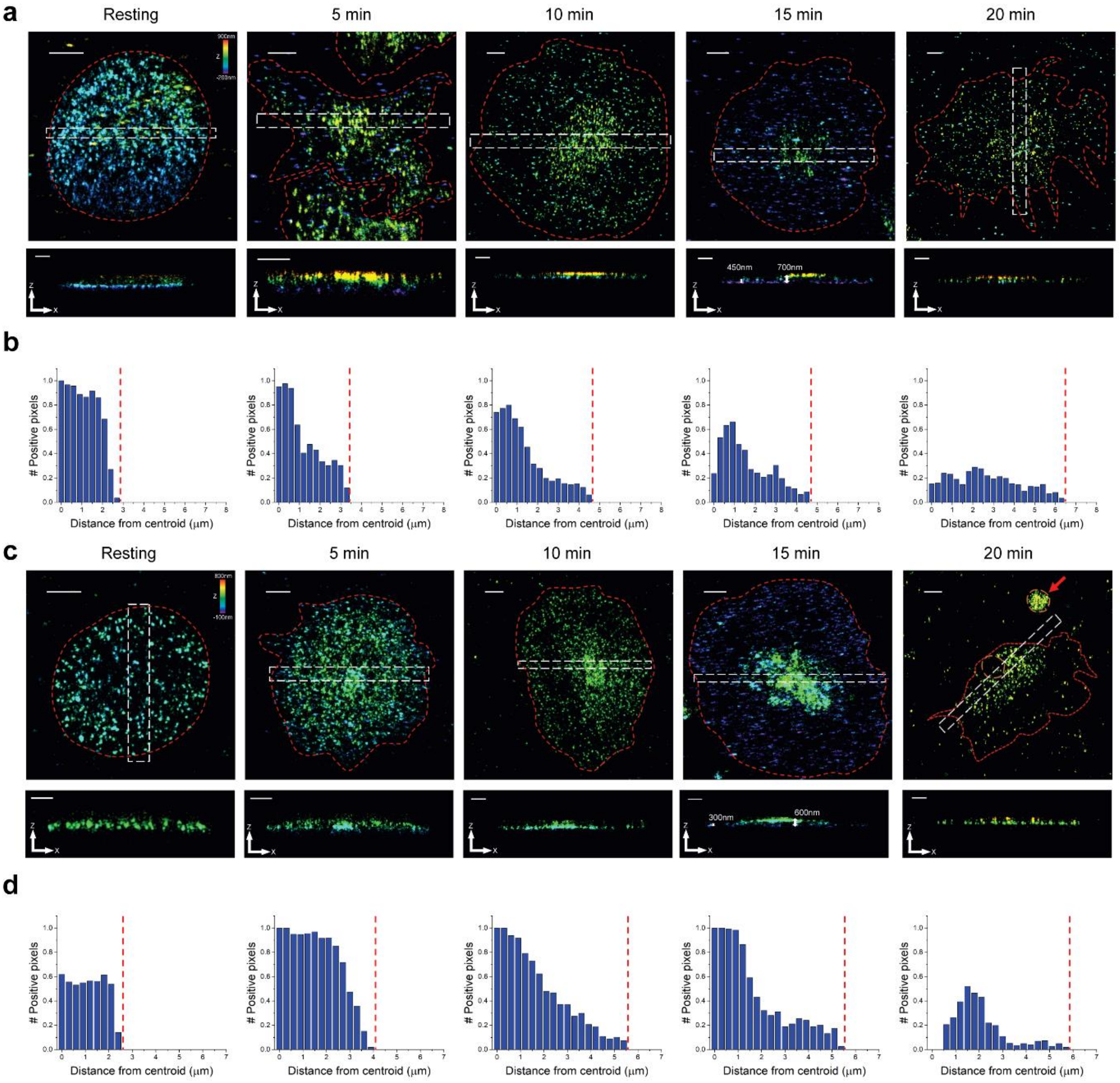

Figure S6. Super-resolution images of other cytoskeletons in the activated platelet.

(a) 3D STORM images of βII-spectrin in the activated platelet. βII-spectrin was observed from the platelets that were fixed at different activation time points (0, 5, 10, 15, 20 min); x-y (top) and x-z (bottom) projections of the white boxed regions are shown. The red dashed line represents the boundary of the platelet identified from the DIC images. (b) The radial distribution graph of βII-spectrin showing how βII-spectrin-positive pixel density varies as a function of distance from the centroid of the platelet shown in (a), implying the centralization of βII-spectrin upon activation until 15 min of treatment. (c) 3D STORM images of vimentin intermediate filaments in the activated platelet. Vimentins were observed from the platelets that were fixed at different activation time points (0, 5, 10, 15, 20 min); x-y (top) and x-z (bottom) projections of the white boxed regions are shown. The red dashed

line represents the boundary of the platelet identified from the DIC images. (d) The radial distribution graph of vimentin showing how vimentin-positive pixel density varies as a function of distance from the centroid of the platelet shown in (c), implying the centralization of βII-spectrin upon activation. Scale bar: 1 μm.

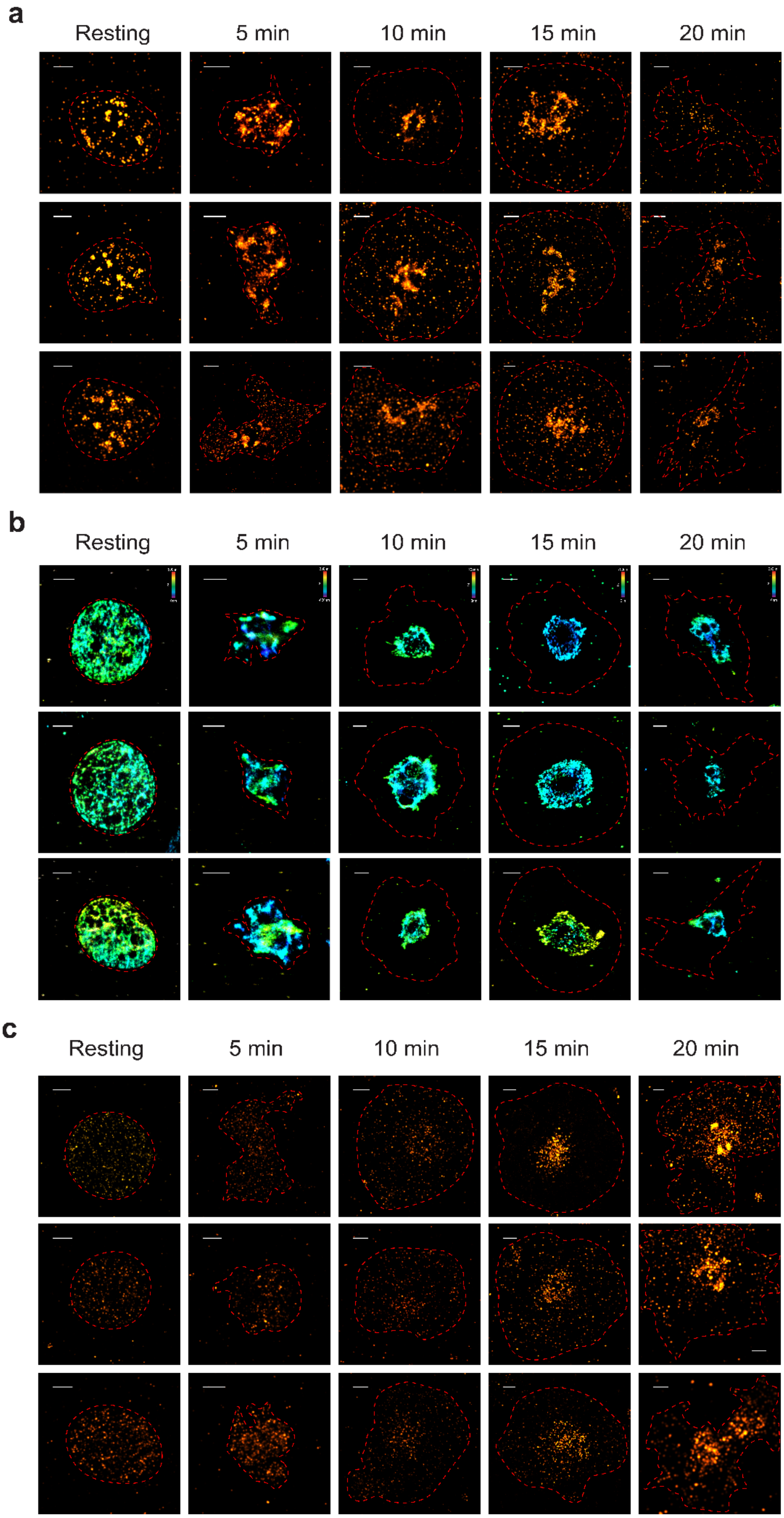
a
Resting
5 min
10 min
15 min
20 min
b
Resting
5 min
10 min
15 min
20 min
c
Resting
5 min
10 min
15 min
20 min

Figure S7.

(a) 2D STORM image examples of mitochondria in activated platelets. (b) 2D STORM image examples of DTS in the activated platelet. (c) 2D STORM image examples of autophagosome in the activated platelet. The red dashed line represents the boundary of the platelet identified from the corresponding DIC. Scale bar: 1 μm.

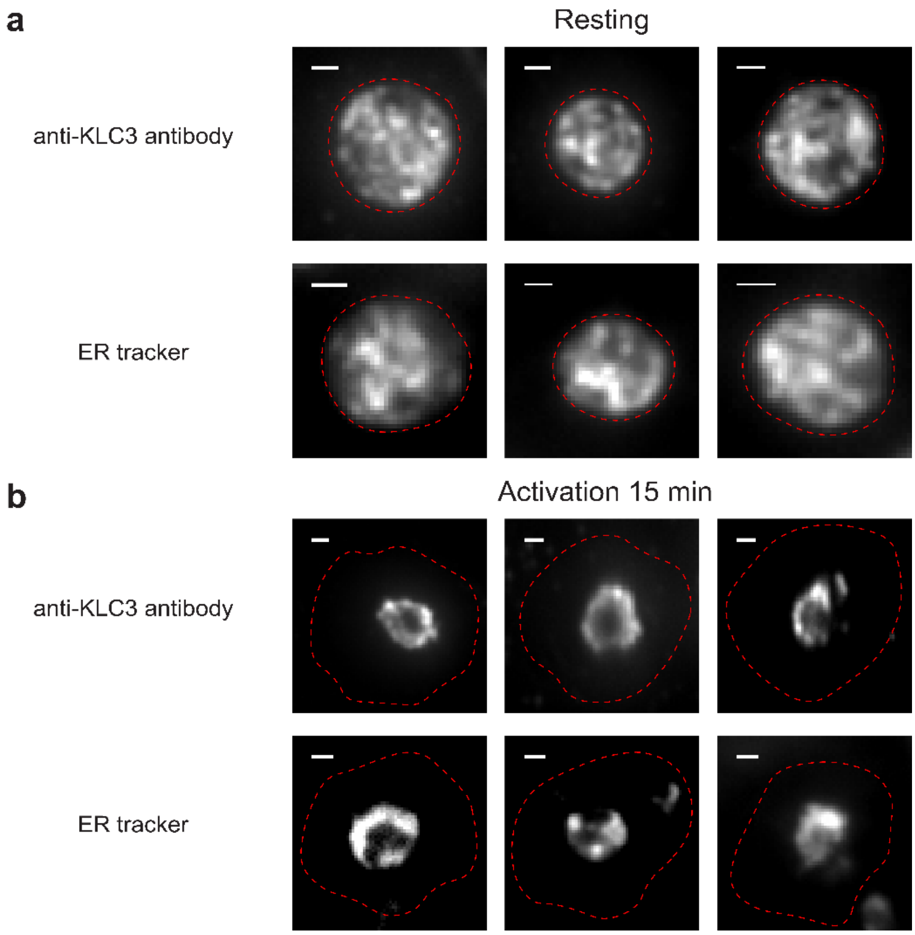

Figure S8.

Fluorescence images of DTS using anti-KLC3 antibody (top) and ER tracker (bottom) for (a) resting and (b) activated platelets. Similar DTS morphologies were observed from the two approaches. Scale bar: 1 μm.

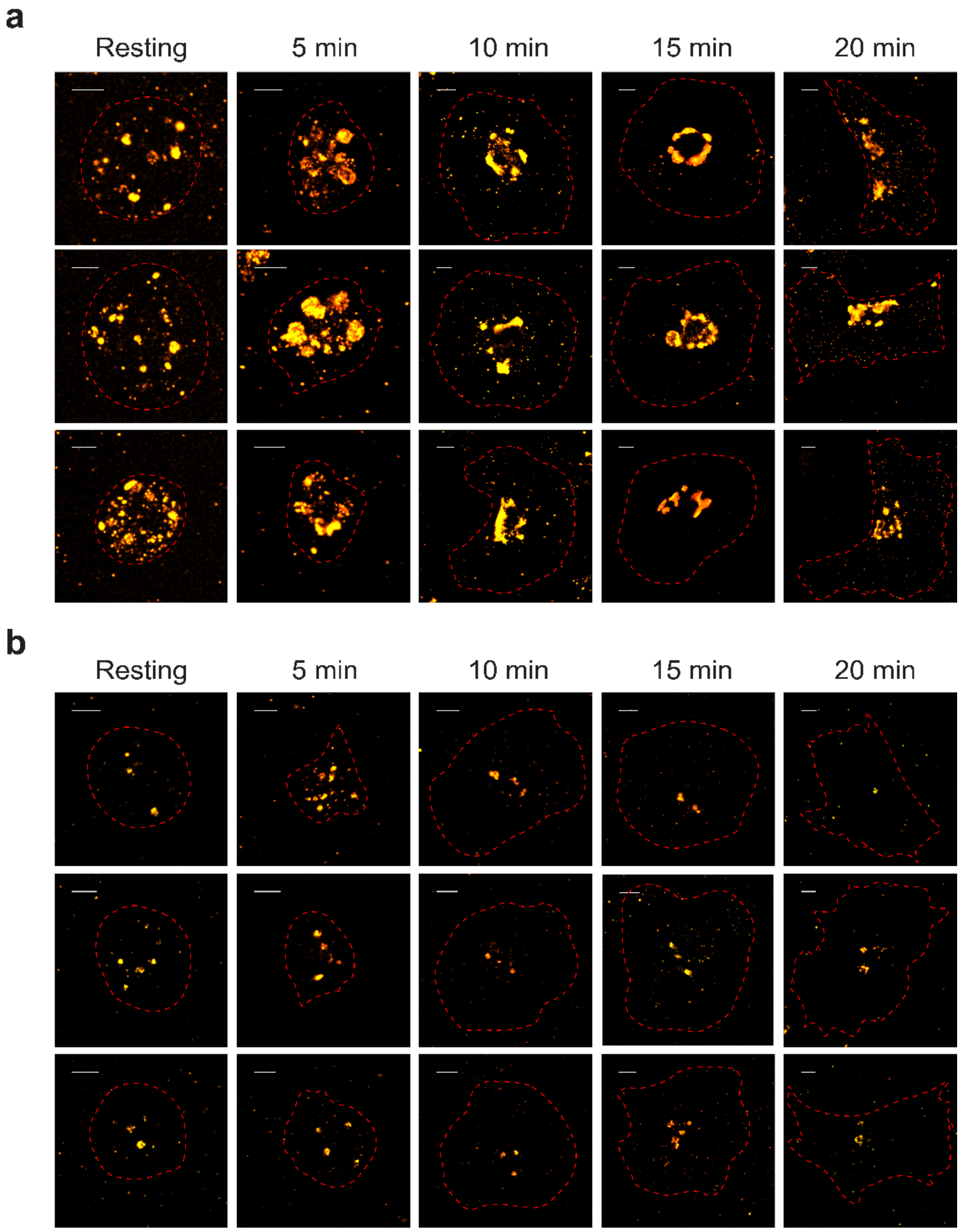

Figure S9.

(a) 2D STORM image examples of α-granules in activated platelets. (b) 2D STORM image examples of dense granules in the activated platelet. The red dashed line represents the boundary of the platelet identified from the corresponding DIC. Scale bar: 1 μm.

Supplementary Note 5

To quantify the α-granule clusters, we set 200 nm diameter of the cluster as the minimum size criterion to distinguish α-granules from other background signals. The average area of α-granules was 0.04 - 0.20 μm^2, which

is consistent with previous results (200 - 400 nm in diameter). To quantify the dense granule clusters, we used 150 nm, the average diameter of a cluster, as the minimum size criteria to distinguish the dense granule from other background signals. Under these criteria, on average, three dense granules were counted per resting platelet, which is consistent with previously reported values (Fig. 6g).

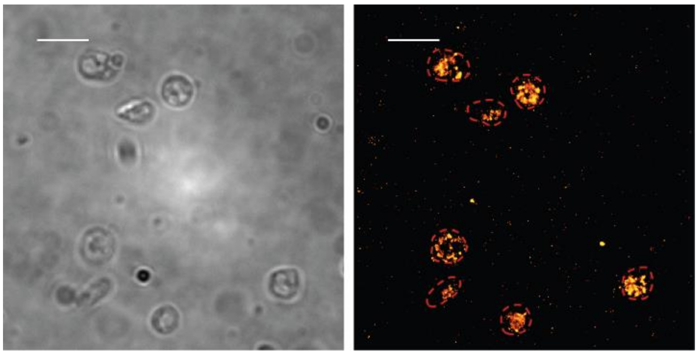

Figure S10.

Representative DIC (left) and 2D STORM (right) images of α-granules in cytochalasin-D-pretreated activated platelets (15 min), which were not spread out at all. Scale bar: 5 μm.

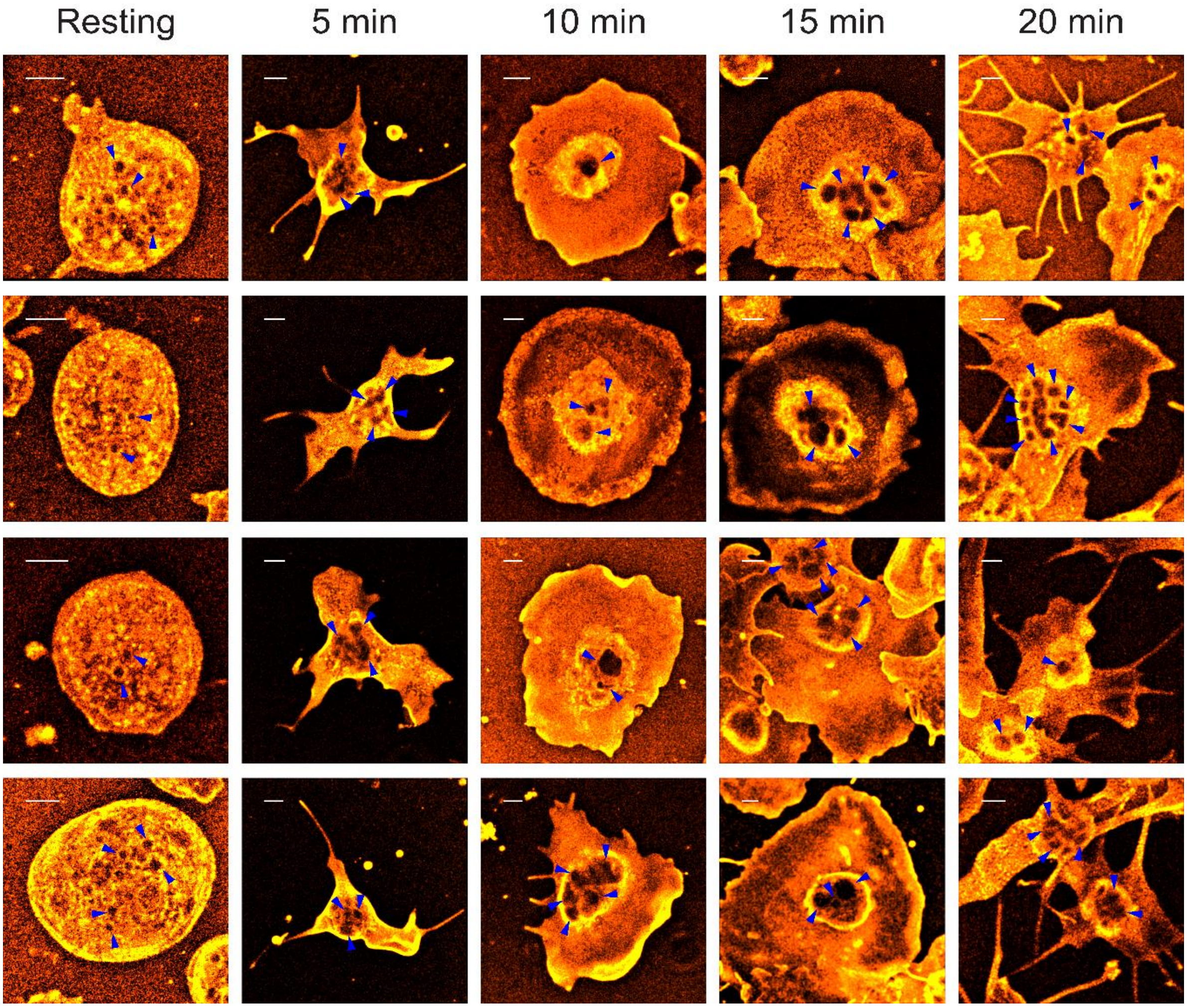

Figure S11.

2D STORM image examples of a Nile Red-labeled activated platelet showing OCS. Blue arrows: OCS. Scale bar: 1 μm.

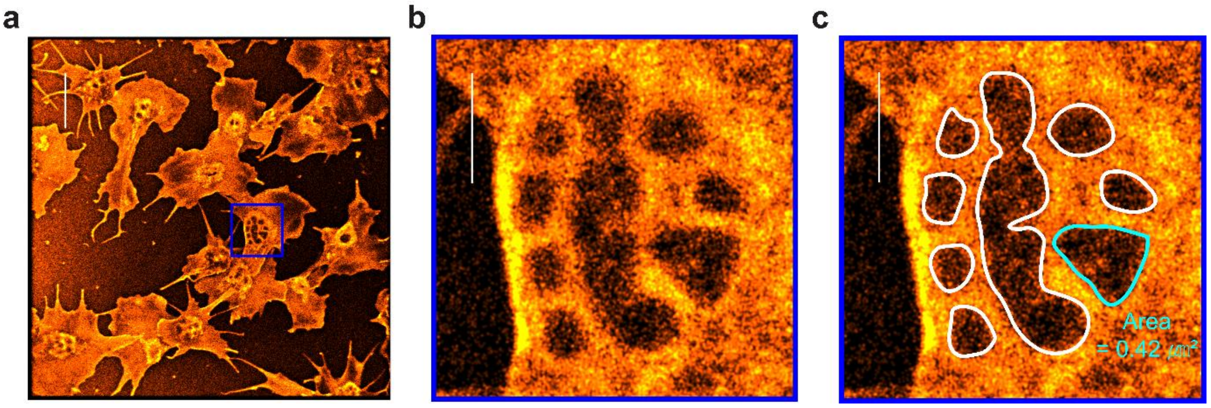

Figure S12.

(a) 2D STORM image of a Nile Red-labeled activated platelet (15 min sample) showing the OCS. (b) Magnified view of the boxed region in (a). (c) Size measurement of the OCS with its identified boundary. Scale bar: 5 μm in (a) and 1 μm in (b).

Supplementary Note 6

For membrane staining, the sample was stained with 100 nM Nile Red solution in DPBS for 20 - 30 min at RT, and immediately imaged using STORM. We tested both permeabilized and non-permeabilized platelets, and found that the permeabilized sample exhibits both of the OCS and the membrane organelles, as reported before[5]. (Fig. S13) Since the permeabilized platelets showed stained membrane organelles more clearly than the OCS, we used the non-permeabilized sample to quantify OCS, which exhibits the OCS more clearly. Scale bar: 1 μm.

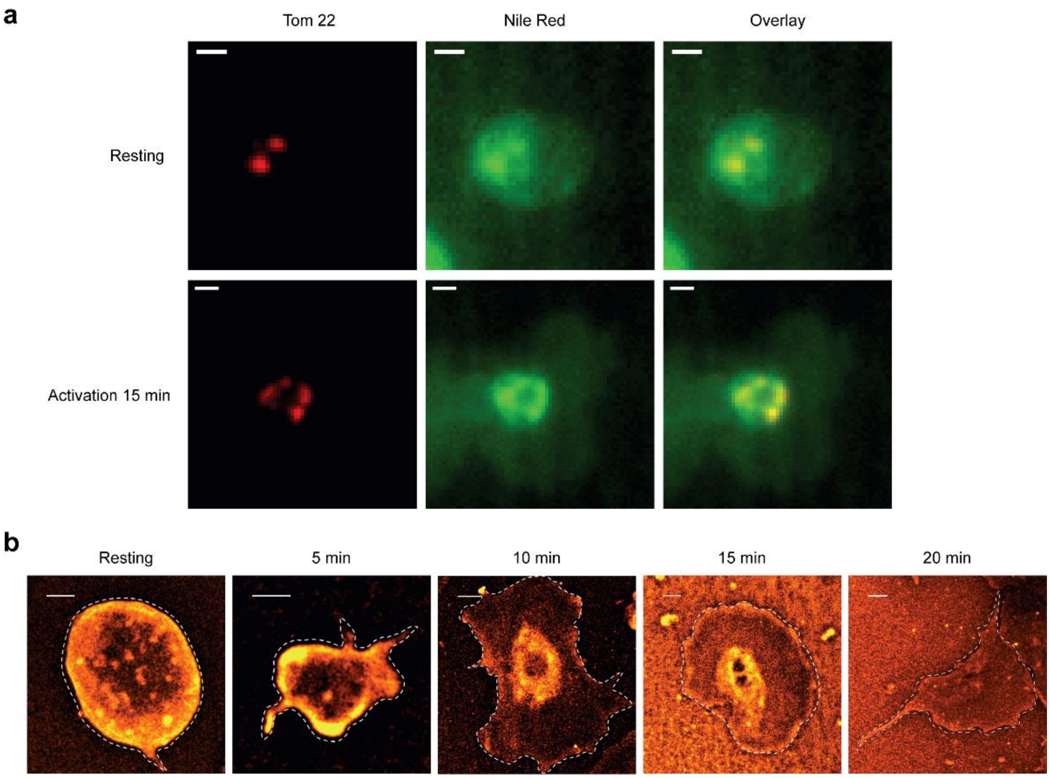

Figure S13.

(a) Two-color-fluorescence images of the permeabilized platelets stained using Nile Red and Tom22 antibody, showing that a Nile Red also stains the mitochondria. (b) 2D STORM images of a Nile Red-labeled permeabilized platelet showing OCS and membrane organelles. They were observed from the platelets that were fixed at different activation time points (0, 5, 10, 15, 20 min). Scale bar: 1 μm.

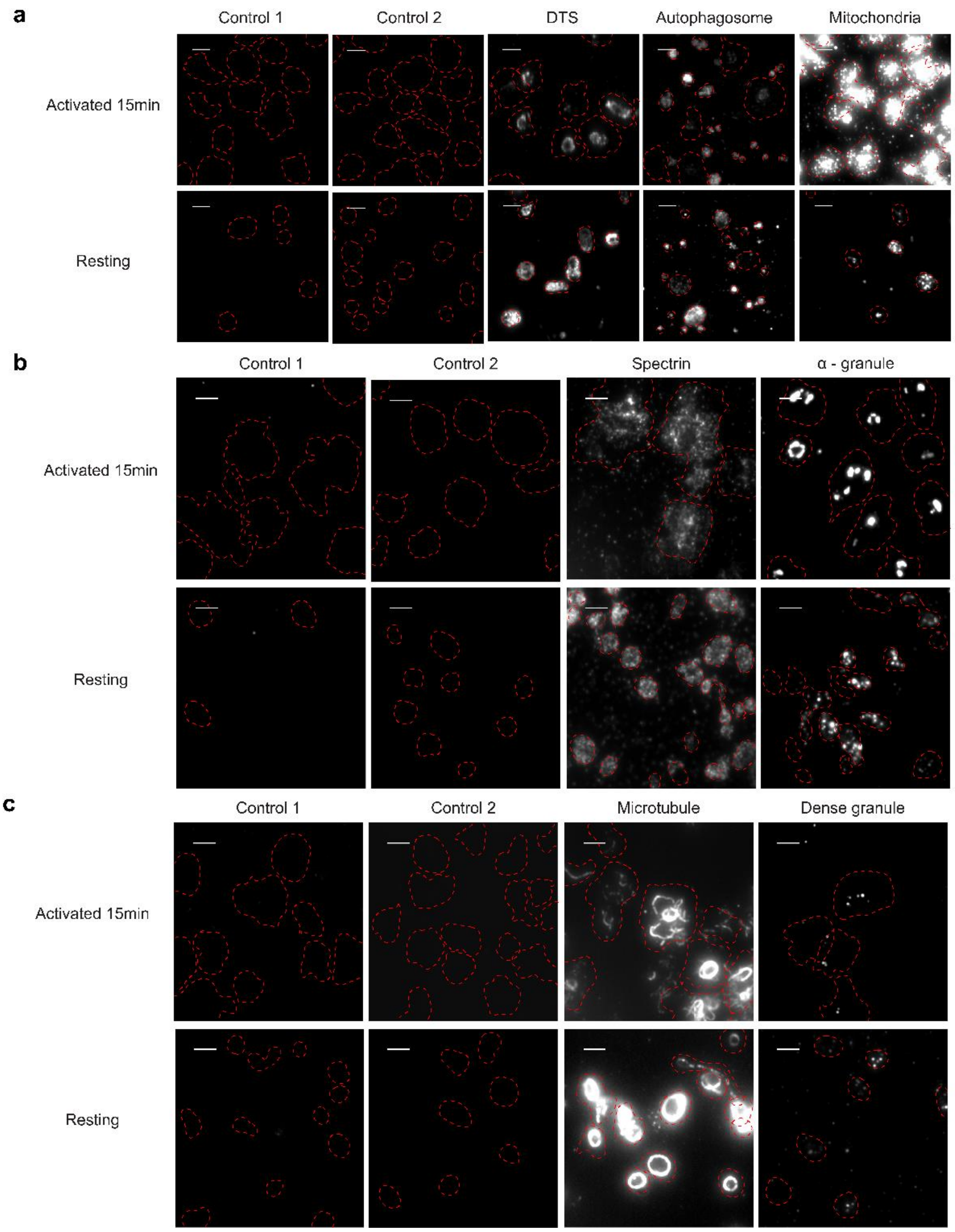

Figure S14. (a) Fluorescence images of control samples for comparison with the fluorescence images of samples labeled with the rabbit primary antibody (DTS, autophagosome, mitochondria) and the Alexa Fluor 647 labeled anti-rabbit secondary antibody under the same contrast level. (b) Fluorescence images of control samples for comparison with the fluorescence images of samples labeled with the mouse primary antibody (spectrin, α-granule) and the Alexa Fluor 647 labeled anti-mouse secondary antibody under the same contrast level. (c) Fluorescence images of control samples for comparison with the fluorescence images of samples labeled with the rat primary antibody (microtubule, dense granule) and the Alexa Fluor 647 labeled anti-rat secondary antibody under the same contrast level. Control 1: Fluorescence images of corresponding isotype control antibody, control 2: Fluorescence images of samples stained only with the secondary antibody without primary antibody to check the non-specific binding of secondary antibodies. Scale bar: 5 μm.

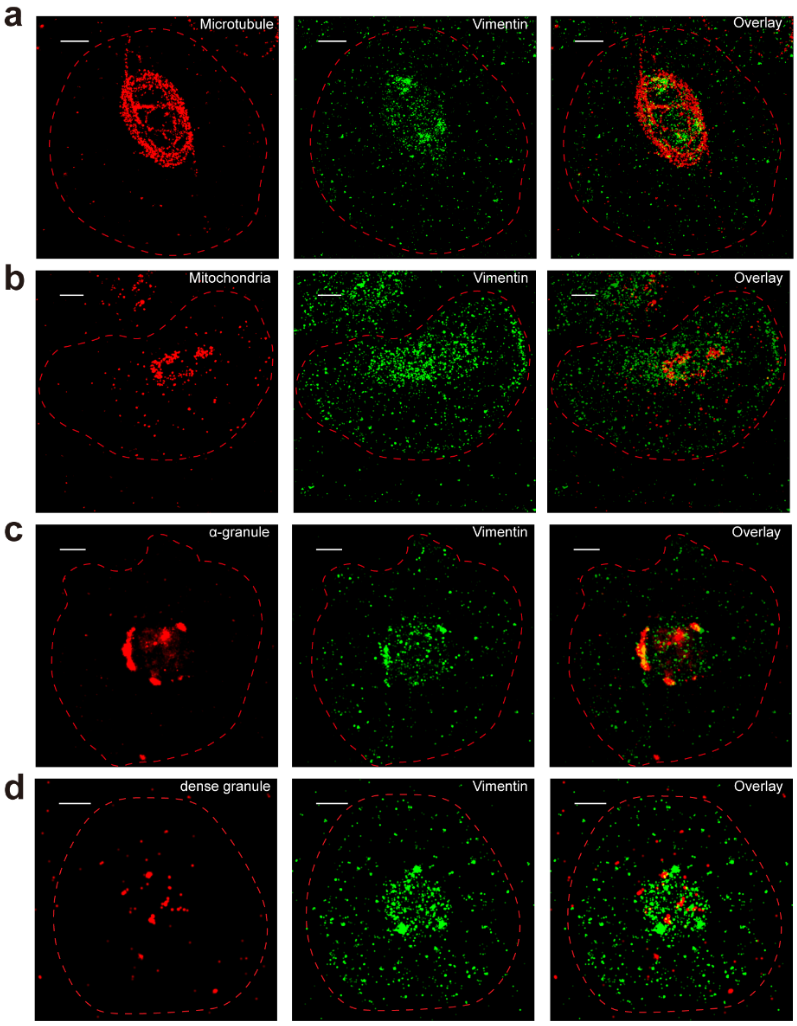

Figure S15. Two-color STORM image of vimentin(green) and (a) microtubules, (b) mitochondria (red), (c) α–granules, and (d) dense granules (red). The red dashed line represents the boundary of the platelet identified from the corresponding DIC images. Scale bar: 1 μm.

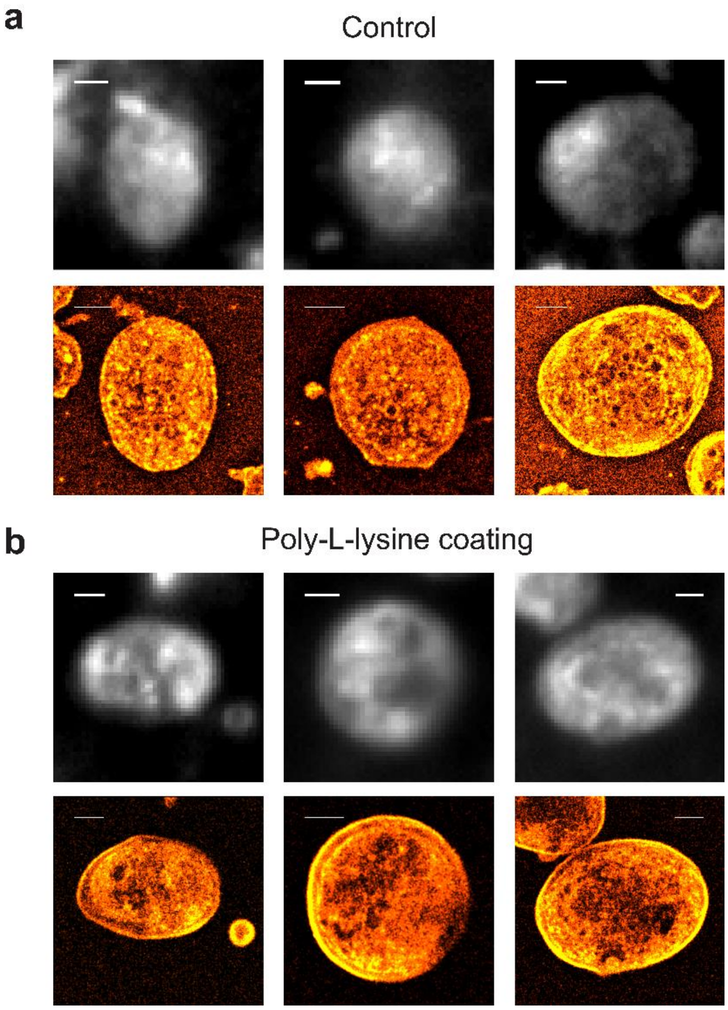

Figure S16. Diffraction-limited (top) and 2D STORM (bottom) images of Nile Red labeled resting platelets showing OCS plated on (a) the bare glass and (b) the poly-L-Lysine coated glass. Scale bar: 1 μm.

Supplementary Video 1.

3D optical diffraction tomography video showing a platelet with dynamic protrusions for sensing the environment as the first step of platelet activation. Scale bar: 1 μm.

Supplementary Note 7

Since the three-dimensional optical diffraction tomography is based on the measurement of three-dimensional refractive index distributions of samples, it clearly reveals these balloons in platelets. (Supplementary Video 2) Although previous attempts to assess ballooning in platelets have been limited by the methods of investigation due to the fragility of the balloon structure, platelets have long been reported to transform to form balloons upon activation.

Supplementary Video 2.

The 3D optical diffraction tomography video showing a platelet undergoing ballooning on adhesion to a glass surface as the beginning step of platelet activation. Scale bar: 1 μm.

Supplementary Video 3.

The 3D reconstructed HV-EM images of the activated platelet using tomographic slices.

Reference

1 Patel-Hett, S. *et al.* The spectrin-based membrane skeleton stabilizes mouse megakaryocyte membrane systems and is essential for proplatelet and platelet formation. *Blood* **118**, 1641-1652, doi:10.1182/blood-2011-01-330688 (2011).

2 Pan, L., Yan, R., Li, W. & Xu, K. Super-resolution microscopy reveals the native ultrastructure of the erythrocyte cytoskeleton. *Cell Rep.* **22**, 1151-1158 (2018).

3 Podor, T. J. *et al.* Vimentin exposed on activated platelets and platelet microparticles localizes vitronectin and plasminogen activator inhibitor complexes on their surface. *J. Biol. Chem.* **277**, 7529-7539, doi:10.1074/jbc.M109675200 (2002).

4 Weyrich, A. S., Schwertz, H., Kraiss, L. W. & Zimmerman, G. A. Protein synthesis by platelets: historical and new perspectives. *Journal of thrombosis haemostasis* **7**, 241-246 (2009).

5 Moon, S. *et al.* Spectrally Resolved, Functional Super-Resolution Microscopy Reveals Nanoscale Compositional Heterogeneity in Live-Cell Membranes. *J. Am. Chem. Soc.* **139**, 10944-10947, doi:10.1021/jacs.7b03846 (2017).
